# Neurodevelopmental outcomes after prenatal exposure to lamotrigine monotherapy in women with epilepsy: a systematic review and meta-analysis

**DOI:** 10.1186/s12884-023-06242-9

**Published:** 2024-02-02

**Authors:** Audrey Peron, Cyndie Picot, Lucie Jurek, Mikaïl Nourredine, Emmanuelle Ripoche, Priscilla Ajiji, Michel Cucherat, Judith Cottin

**Affiliations:** 1 Service Hospitalo-Universitaire de Pharmacotoxicologie de Lyon, Hospices Civils de Lyon, Bât. A-162, avenue Lacassagne, Lyon Cedex 03, 69424 France; 2 Pôle de psychiatrie de l’Enfant et l’Adolescent, Centre Hospitalier Le Vinatier, Bron, France; 3 Service Universitaire d’Addictologie de Lyon, Centre Hospitalier Le Vinatier, Bron, France; 4 Adverse Events and Incidents Department-Surveillance Division, Agence nationale de sécurité du médicament et des produits de santé (ANSM), Saint Denis, France; 5 Faculté de Santé, Université Paris-Est Créteil, Créteil, EA 7379 France

**Keywords:** Lamotrigine, Systematic review, Meta-analysis, Pregnancy, Neurodevelopmental disorders, Epilepsy

## Abstract

**Background:**

Lamotrigine has become one of the most commonly prescribed antiseizure medications (ASM) in epileptic women during pregnancy and therefore requires regular updates regarding its safety. The aim of this study was to estimate the association between *in utero* exposure to lamotrigine monotherapy and the occurrence of neurodevelopmental outcomes.

**Methods:**

All comparative studies assessing the occurrence of neurodevelopmental outcomes after epilepsy-indicated lamotrigine monotherapy exposure during pregnancy were searched. First, references were identified through a snowballing approach, then, through electronic databases (Medline and Embase) from 2015 to June 2022. One investigator evaluated study eligibility and extracted data and a second independent investigator reviewed the meta-analysis (MA). A systematic review and random-effects model approach were performed using a collaborative WEB-based meta-analysis platform (metaPreg.org) with a registered protocol (osf.io/u4gva).

**Results:**

Overall, 18 studies were included. For outcomes reported by at least 4 studies, the pooled odds ratios and 95% confidence interval obtained with the number of exposed (N1) and unexposed children (N0) included were: neurodevelopmental disorders as a whole 0.84 [0.66;1.06] (N1 = 5,271; N0 = 22,230); language disorders or delay 1.16 [0.67;2.00] (N1 = 313; N0 = 506); diagnosis or risk of ASD 0.97 [0.61;1.53] (N1 = at least 5,262; N0 = 33,313); diagnosis or risk of ADHD 1.14 [0.75;1.72] (N1 = at least 113; N0 = 11,530) and psychomotor developmental disorders or delay 2.68 [1.29–5.56] (N1 = 163; N0 = 220). The MA of cognitive outcomes included less than 4 studies and retrieved a significant association for infants exposed to lamotrigine younger than 3 years old but not in the older age groups.

**Conclusion:**

Prenatal exposure to lamotrigine monotherapy is not found to be statistically associated with neurodevelopmental disorders as a whole, language disorders or delay, diagnosis or risk of ASD and diagnosis or risk of ADHD. However, the MA found an increased risk of psychomotor developmental disorders or delay and cognitive developmental delay in less than 3 years old children. Nevertheless, these findings were based exclusively on observational studies presenting biases and on a limited number of included children. More studies should assess neurodevelopmental outcomes in children prenatally exposed to lamotrigine.

**Supplementary Information:**

The online version contains supplementary material available at 10.1186/s12884-023-06242-9.

## Background

There is a growing interest for neurodevelopmental consequences after *in utero* exposure to antiseizure medications (ASM). However, long-term outcomes are rarely addressed in pregnancy safety studies [1]. Although a higher occurrence of adverse neurodevelopmental outcomes has been identified for valproate [1, 2] and more recently for topiramate [3], the risks for other ASM remain unclear or have not yet been assessed [1, 4]. In the last few decades, these specific concerns, as well as the risk of teratogenicity, has led to changes in prescription practices from older to newer ASM. Lamotrigine has thus been increasingly prescribed in pregnant women worldwide and is now the most frequently used ASM along with levetiracetam [5, 6]. Although there is extensive data on the malformative risk associated with lamotrigine use [7–10], there is still a lack of evidence regarding neurodevelopmental outcomes in the scientific literature.

In 2017, for the first time, a network meta-analysis (MA) [2] raised concerns about *in utero* exposure to lamotrigine and the risk of autism diagnoses. Since the release of this network MA, a large number of new studies investigating neurodevelopmental outcomes have been published [3, 11–18]. These concerns therefore need to be reassessed.

The purpose of the present work was to update the knowledge on the neurodevelopmental consequences of prenatal exposure to lamotrigine monotherapy in women with epilepsy by means of a systematic review and MA.

## Methods

A systematic review and MA were conducted to assess the association between *in utero* exposure to lamotrigine monotherapy and the occurrence of neurodevelopmental disorders. This study was reported in accordance with the standards of the Cochrane collaboration [19, 20] using MOOSE (Meta-analyses Of Observational Studies in Epidemiology) and PRISMA (Preferred Reporting Items for Systematic Reviews and Meta-Analyses) guidelines when appropriate.

Data management and analyses were conducted using metaPreg (metaPreg.org), a proprietary collaborative WEB-based MA platform. The master protocol was established before starting the study, was registered in open science framework (osf.io/u4gva), is available on the metaPreg website (http://metapreg.org/doc/protocol.pdf) and was published in a peer-reviewed journal [21].

### Study identification

A therapeutic class approach was used in order to improve comprehensiveness. All drugs of the antiepileptic class were searched to also identify studies that included lamotrigine but that did not index it individually. The relevant studies were identified by a two steps process: (i) through a snowballing approach to identify relevant papers based on the reference lists of published meta-analyses and/or systematic reviews; (ii) with the search of two electronic databases (Medline and Embase) starting from the last publication search date of the most recent MA [2] (i.e. 2015) until June 2022 (Supplement S1).

### Criteria for considering studies

All studies with a non-treated comparator group, assessing the association between lamotrigine monotherapy use in pregnant women with epilepsy and neurodevelopmental outcomes in offspring were eligible. Studies assessing several antiseizure medications, without non-treated comparator, were excluded because there is no antiseizure medication with sufficient reassuring data to be used as an active comparator.

#### Types of studies

Prospective cohort studies, historical cohort studies (also known as retrospective cohort studies), case–control studies, and randomized clinical trials were included. Studies were included regardless of publication status or language of publication. In case of iterative studies using the same database, only the most recent publication was included. In case of overlapping data (different publications using the same dataset, on the same study period, to assess the same outcome), only the one with the largest sample size or with a methodology that provided a better consideration of the confounding factors was kept. If a new study overlaps partially the previous one, the both were included only if the majority of the information is not common, i.e. the new study overlaps less than 50% of previous study period.

#### Types of exposure

The exposure was restricted to monotherapy in order to clearly identify the contribution of lamotrigine and avoid confounding by other ASM. Secondly, only pregnancies exposed to lamotrigine for an epilepsy indication were included to maintain a relative homogeneity in terms of confounding factors (notably comorbidities) and in the doses used, which may differ according to the indication; when the indication was not specified, the study was not included. No selection was made on the period of exposure during pregnancy, because no specifically relevant exposure period has been previously identified.

#### Types of outcomes

The neurodevelopmental outcomes of interest were: neuro-developmental disorders considered as a whole by authors (i.e. including several cognitive and behavioral disorders as a whole); language disorders or delay; psychomotor developmental disorders or delay; diagnosis or risk of attention deficit / hyperactivity disorder (ADHD); diagnosis or risk of autism spectrum disorder (ASD); cognitive developmental delay (at < 3 years old, 3–6 years old, and > 6 years old); severe cognitive developmental delay (at 3–6 years old and > 6 years old); and learning disorders. The MA for each outcome of interest are presented but the sensitivity analyses, risk-of-bias assessment, and publication bias assessment were only performed for the outcomes reported by at least four studies.

Since neurodevelopmental disorders cover a wide range of outcomes assessed by numerous scales and tools, a correspondence matrix was developed with the help of a child psychiatrist and a psychiatrist to aggregate the different measurement tools available with as much clinical relevance as possible (Supplement S2).

If the same neurodevelopmental outcome was assessed for several ages in the same children, the results obtained at the oldest age were preferred (and thus used for the MA) because the disorder could be considered as better established [22, 23]. Moreover, if the same outcome was assessed using different scales, the results obtained with the most reliable one were used (for example, a confirmed clinical diagnosis > tests performed by practitioners > parent-reported screening tools).

### Study selection

Study selection was a two-stage process. First, the abstracts of all the studies identified in the above search were screened by one reviewer (AP) assisted by automation tools based on artificial intelligence (metaPreg.org) [24]. Then, the full-text reports of potentially relevant studies were assessed by the same reviewer (AP), to establish whether they fulfilled the inclusion criteria. In case of doubt regarding the inclusion of a study, the matter was discussed with the scientific directors of the project (JC, CP, and MC) until agreement was reached. The process of study selection was documented and reported using a PRISMA flow diagram. The feasibility and acceptance of the semi-automated process and single-screening approach were assessed and globally, it was shown that this process reduces the time required for a MA without altering expert confidence in the methodological and scientific rigor [24, 25].

### Data extraction and analyses

Information regarding study description, methods, and results were extracted from the included studies using a standardized electronic data collection form on a WEB-based collaborative MA platform (metaPreg.org) (Supplement S3). Authors were contacted if information was missing or unclear.

When available, the adjusted effect measures (odds ratio (OR), risk ratio, or hazard ratio) from the included studies were used for the analyses. When not available, the crude ORs were used. If the study did not report any OR, they were calculated with the raw data provided by the authors. Cognitive assessments scored with dichotomous criteria were preferred, otherwise, continuous scales were considered and transformed to an OR according to the formula recommended by the Cochrane handbook [26].

Some studies considered different comparator groups and provided estimates for each. In the main analysis, the type of control group was chosen in the following preferred order: (i) pregnant women with epilepsy or history of epilepsy not exposed to ASM during pregnancy (but that could have received treatment prior to pregnancy), referred to as “unexposed, women with epilepsy”; (ii) pregnant women not exposed to ASM but whose health status (presence or absence of disease) was unspecified or not known, called “unexposed (general population or not otherwise specified (NOS))”; and (iii) pregnant non-epileptic women not exposed to ASM, defined as “unexposed, disease free”.

Three sensitivity analyses according to (i) the type of study (cohort or case-control), (ii) the type of comparison group (“unexposed, women with epilepsy” or “unexposed, disease free or unspecified”), and (iii) adjustment performed by the included studies (Yes/No) were undertaken to investigate sources of heterogeneity for the outcomes reported by at least four studies.

After completing the MA, a quality control process was conducted by a second independent reviewer (CP) who checked every item, including missing details. Disagreements were discussed among the biocurators until resolution or during a project meeting.

### Assessment of risk of bias in included studies

For outcomes reported by at least four studies, the risk of bias of the included studies was assessed and presented at an outcome-level using the Cochrane Risk of Bias Tool for Non-Randomized Studies of Interventions (ROBINS-I) [27]. The ROBINS-I tool signaling questions were adapted to observational studies evaluating medicine safety in pregnancy. Six types of bias were considered: (a) selection bias, (b) confounding bias, (c) bias in classification of exposure, (d) bias due to missing data, (e) bias in measurement outcomes, and (f) bias in selection of reported results. The ROBINS-I item on bias due to deviations from intended interventions was considered to be specific to randomized clinical trials and not applicable to observational studies. For confounding bias, four levels of risk were considered: low, moderate, serious, and critical. For the other biases, only three levels of risk were considered (low, moderate, and critical) as no situation where a degree of fineness between critical and serious was identified. For each bias type, an additional unclear category was available, when the data reported was insufficient to allow assignment to the aforementioned categories.

One biocurator (AP) performed the assessment and if any doubts occurred, they were resolved by consensus during a multidisciplinary team meeting. The studies were not excluded on the grounds of risk of bias.

### Statistical analysis

The data from observational studies were used to perform random-effects MA. The summary effect size with a pooled OR and its 95% confidence interval (95% CI) was estimated using the inverse variance method based on the DerSimonian and Laird random-effects model. The MA was performed by using only the summary data and no attempt was made to obtain individual patient data. Forest plots were produced using the meta package of the R language for outcomes reported by at least four studies.

The assessment of heterogeneity was performed by means of the I-squared statistic [28] and tau-squared test. The random-effects model was selected *a priori*, whatever the heterogeneity, in order to consider both within-study and between-study variation by incorporating the heterogeneity of effects into the overall analyses. The publication bias and small study effect were assessed using the funnel plot for outcomes reported by at least four studies and using the Egger’s test [29] when at least ten studies were included. The trim and fill method was used to determine the number of missing studies and to adjust for publication bias [30].

All the data used in this work are stored in the collaborative WEB-based MA platform (metaPreg.org) and available on request.

## Results

**Fig. 1:**
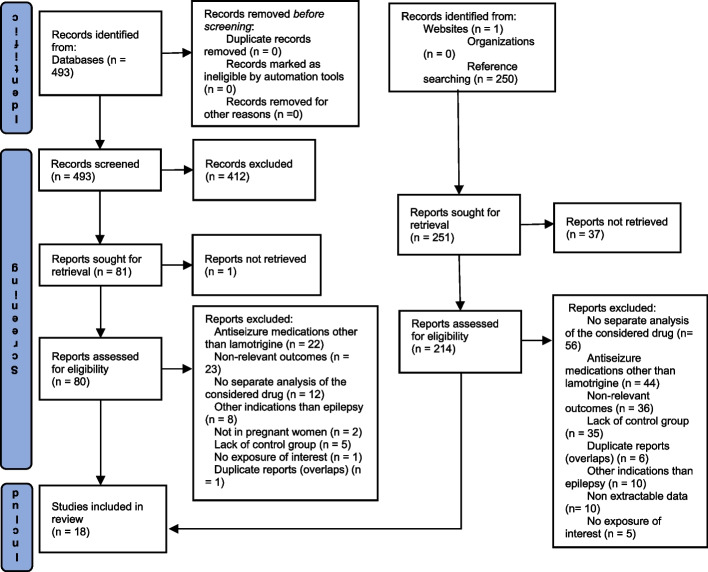
PRISMA flow diagram for selection of included studies

Overall, 744 records were screened, 493 of which were identified through the literature search of electronic databases and 251 from other sources (previously published MA and websites). Of the 294 full-text reports assessed for eligibility, 18 studies [3, 11–18, 31–39] concerned neurodevelopmental disorders after *in utero* exposure to lamotrigine monotherapy in women with epilepsy and were included in the MA (Fig. 1).

The other full-text reports mainly documented other outcomes, other indications, an ASM other than lamotrigine, or were duplicates (Supplement S4). The details of the 18 included studies are available in Table 1.

**Table 1:** Overview of the main characteristics of included studies

First author, year (+ overlap)	Setting / source of data	Nb of participants	Maternal age at pregnancy in years, mean (SD)	Maternal epilepsy type	Convulsive seizures during pregnancy	Outcome	Adjusted covariates
Baker, 2015 [31] (overlap: Bromley, 2010 [32])	11 National Health Service hospitals within Merseyside and Greater Manchester (LMNDG group), UK	Exposed = 36	27.7 (6)	Idiopathic generalized epilepsy 23.3% Focal epilepsy 56.7% Unclassified 20.0%	40.0%	- Language (based on continuous data) - Cognitive developmental delay (based on continuous data)	
		Unexposed WWE = 34	25.9 (5)	Idiopathic generalized epilepsy 32.0% Focal epilepsy 40.0% Unclassified 28.0%	4.0%		None
		Unexposed disease free = 287	29.4 (5)				
Bjørk, 2018 [11] (overlap: Veiby, 2013 [38])	Norwegian Mother and Child Cohort Study (MoBa) and The Medical Birth Registry of Norway	Exposed = 104	NA	NA	NA	- ASD	
		Unexposed WWE = 389	NA	NA	NA		None
		Unexposed disease free = 104,222	NA				
Bjørk, 2022 [3]	SCAN-ASD: Nordic register-based study of antiepileptic drugs in pregnancy	Exposed = 5073	30.1 (5.2)^a^	NA	NA	- Neuro-developmental disorders - ASD - Cognitive developmental delay	
		Unexposed WWE = 21,634	29.6 (5.4)	NA	NA		Maternal age, education and marital status, parity, child’s birth year, sex, and country of birth. All the models were run with separate strata for country and year. Maternal use of antidepressants or opioids, depression, anxiety, personality disorders, number of chronic somatic diseases, and number of hospitalizations the year before last menstrual period were sometimes added to the models.
		Unexposed NOS = 4,463,879	30.2 (5.2)	NA	NA		None
Bromley, 2013 [39] (overlap: Baker, 2015 [31], Charlton 2017 [12], Bromley, 2010 [32], and Bromley, 2008 [40])	11 National Health Service hospitals within Merseyside and Greater Manchester (LMNDG group), UK	Exposed = 36	28 (NA)	Idiopathic generalized epilepsy 23.3% Focal 56.7% Unclassified 20.0%	40.0%	- Neuro-developmental disorders - ASD - ADHD - Psychomotor	
		Unexposed WWE = 34	26 (NA)	Idiopathic generalized epilepsy 32.0% Focal 40.0% Unclassified 28.0%	4.0%		None
		Unexposed disease free = 285	29 (NA)				Seizures during pregnancy, maternal IQ, maternal age, socio-economic status, alcohol or nicotine exposure, gender and gestational age at birth. However, the variables kept in the final regression model are not specified (data not shown).
Bromley, 2010 [32]	11 National Health Service hospitals within Merseyside and Greater Manchester (LMNDG group), UK	Exposed = 34	NA	NA	NA	- Cognitive developmental delay	
		Unexposed WWE = 27	NA	NA	NA		None
		Unexposed disease free = 230	NA				Matched for age, within a 5-year band, and for parity
Charlton, 2017 [12]	The United Kingdom Clinical Practice Research Datalink (CPRD)	Exposed = 122	NA	NA	NA	- Neuro-developmental disorders	
		Unexposed WWE = 472	NA	NA	NA		
		Unexposed disease free = 6048	28.9				Matching on maternal age at pregnancy start, year of delivery, sex of the child, general practitioner practice/socioeconomic status of general practitioner practice, and adjustment for alcohol drinking status.
Cohen-Israel, 2018 [13]	The Beilinson Teratology Information Service, Israel	Exposed = 83	NA	NA	NA	- Language - ASD - Psychomotor - Learning disorders - Cognitive developmental delay	
		Unexposed disease free = 83	NA				Matched for gestational age and date of birth
Dean, 2007 [34]	Aberdeen Maternity Hospital, Scotland	Exposed = 4	NA	NA	NA	- Neuro-developmental disorders	
		Unexposed WWE = 46	NA	NA	NA		None
Cummings, 2011 [33]	The UK epilepsy and pregnancy register	Exposed = 35	Range: 16–39	NA	NA	- Cognitive developmental delay	
		Unexposed disease free = 44	Range: 20–49				Confounding variables which persisted as significant adverse factors, were age, gender and socioeconomic status.
Elkjaer, 2018 [35]	Danish national registries (the Danish Medical Birth Registry…)	Exposed = 243	NA	Focal 23.5% Generalized 22.2% Other 54.3%	NA	- Learning disorders (based on continuous data)	
		Unexposed WWE = 1909	NA	NA	NA		Test year, child sex, and maternal education and household income at birth.
Gopinath, 2015 [36]	The Kerala Registry of Epilepsy and Pregnancy (KREP), India	Exposed = 1	NA	NA	NA	- Cognitive developmental delay	
		Unexposed WWE = 16	NA	NA	NA		None
Husebye, 2020 [14]	Norwegian Mother and Child Cohort Study (MoBa) and The Medical Birth Registry of Norway	Exposed = 112	29.0 (24.0)^a^	NA	12%	- Language	
		Unexposed WWE = 388	29.0 (25.0)	NA	3%		None
		Unexposed disease free = 113,674	30.0 (39.0)				Maternal age, parental socioeconomic status, low household income, parity, maternal prepregnancy, BMI, maternal report of familial language delay (5y model), smoking and alcohol (8y model), maternal anxiety/depression, Apgar score at 5 min, gestational age, report of seldom/never helping child read letters and sounds during a typical week (5y model) or report of never reading to their child (8y model)
Kasradze, 2017 [15]	The Georgian branch of the International Registry of Antiepileptic Drugs and Pregnancy (EURAP)	Exposed = 3	30.5 (4.9)^a^	Focal 86% Generalized 14%	NA	- Language (based on continuous data) - Cognitive developmental delay (based on continuous data)	
		Unexposed disease free = 50	31.7 (5.2)				Age and gender-matched.
Meador, 2021 [16]	The Maternal Outcomes and Neurodevelopmental Effects of Antiepileptic Drugs (MONEAD) study, US	Exposed = 97	30.9 (5.2)^a^	Generalized 33.8% Focal 59.0% Unclassified/multiple 7.1%	mean (95% CI) 0.7 (0.4, 1.0)	- Language (based on continuous data)	
		Unexposed disease free = 106	29.8 (5.2)				Mother’s IQ, education level, post-birth average Beck Anxiety Inventory (BAI) score, child’s sex, ethnicity, and birthweight.
Rihtman, 2013 [37]	The Israeli Teratogen Information Service	Exposed = 42	33.56 (4.58)	Grand mal 53% Focal/temporal lobe 5.5% Petit mal 14% Other 22% Not reported 5.5%	NA	- Neuro-developmental disorders - ADHD (based on continuous data) - Psychomotor (based on continuous data) - Language (based on continuous data) - Cognitive developmental delay (based on continuous data)	
		Unexposed NOS = 52	33.00 (4.69)	NA	NA		None
Veiby, 2013 [38]	Norwegian Mother and Child Cohort Study (MoBa) and The Medical Birth Registry of Norway	Exposed = 104	NA	NA	NA	- ADHD	
		Unexposed WWE = 393	NA	NA	NA		None
		Unexposed disease free = 107,597	NA				None
Videman, 2016 [17]	The Helsinki University Hospital, Finland	Exposed = 8	NA	NA	NA	- Language (based on continuous data) - Psychomotor (based on continuous data) - Cognitive developmental delay (based on continuous data)	
		Unexposed disease free = 67	NA				None
Wiggs, 2020 [18]	Swedish Medical Birth Register	Exposed = NA	NA	NA	NA	- ASD - ADHD	
		Unexposed WWE = 11,298	NA	NA	NA		Maternal and paternal characteristics included age, highest education, cohabitation status, country of origin, neighborhood deprivation and averaged maternal and paternal disposable income, parental psychiatric and behavioral problems, inpatient diagnosis of seizures in the year before pregnancy, birth order, child sex, and maternal smoking during pregnancy and concurrent medication use.
When result is expressed in a continuous manner in the publication so there is not a countable number of cases, thus indicated as NA in the table							
*ASD* Autism spectrum disorder, *ADHD* Attention deficit / hyperactivity disorder, *NA* Not available, *IQ* Intelligence quotient, *BMI* Body mass index, *WWE* Women With Epilepsy							
^a^When the mean maternal age is only available for all exposures assessed (not only lamotrigine-exposed women)							

Among the considered outcomes, the following were reported by at least four studies: overall neurodevelopmental disorders (defined as several cognitive and behavioral disorders as a whole, without distinction between the disorders), language disorders or delay, psychomotor developmental disorders or delay, diagnosis or risk of ASD, and diagnosis or risk of ADHD. The other outcomes were reported by less than four studies: cognitive developmental delay (at < 3 years old, 3–6 years old, and > 6 years old); severe cognitive developmental delay (at 3–6 years old and > 6 years old); and learning disorders.

### Meta-analyses of neurodevelopmental disorders reported by at least four studies

An overview of the MA results for each outcome reported by at least four studies are presented in Table 2.

**Table 2:** Overview of MA results for all neurodevelopmental outcomes after *in utero* exposure to lamotrigine monotherapy in women with epilepsy

Outcomes	Number of included studies per type of comparator group	Sample size (exposed cases/total exposed) (unexposed cases/total unexposed)	OR and 95% CI	I²; tau²
Neuro-developmental disorders as a whole	*n* = 4 unexposed, WWE; *n* = 1 unexposed (not otherwise specified)	84/5271 456/22,230	0.84 [0.66;1.06]	0%; 0
Language disorders or delay	*n* = 2 unexposed, WWE; *n* = 4 unexposed, disease free; *n* = 1 unexposed (not otherwise specified)	15^a^/313 50^a^/506	1.16 [0.67;2.00]	58%; 0.29
Psychomotor developmental disorders or delay	*n* = 2 unexposed, disease free; *n* = 1 unexposed, WWE; *n* = 1 unexposed (not otherwise specified)	1^a^/163 2^a^/220	**2.68 [1.29;5.56]**	9%; 0.07
Diagnosis or risk of ADHD	*n* = 3 unexposed, WWE; *n* = 1 unexposed (not otherwise specified)	3^a^/ 113^a^ 4^a^/11,530	1.14 [0.75;1.72]	0%; 0
Diagnosis or risk of ASD	*n* = 4 unexposed, WWE; *n* = 1 unexposed, disease free	63^a^/5262^a^ 358^a^/33,313	0.97 [0.61;1.53]	30%; 0.08
	*n* = 2 unexposed, WWE; *n* = 1 unexposed, disease free	50/5186 268/21,743	0.81 [0.59;1.11]	0%; 0
Cognitive developmental delay (< 3 years old)	*n* = 1 unexposed, WWE; *n* = 1 unexposed, disease free	5^a^/42 2^a^/86	**3.42 [1.17;9.98]**	0%; 0
Cognitive developmental delay (3–6 years old)	*n* = 2 unexposed, disease free; *n* = 1 unexposed (not otherwise specified)	1^a^/79 2^a^/146	3.39 [0.56;20.57]	68%; 1.70
Cognitive developmental delay (> 6 years old)	*n* = 2 unexposed, WWE; *n* = 1 unexposed, disease free	12^a^/113 30^a^/124	0.75 [0.41;1.37]	0%; 0
Severe cognitive developmental delay (Mental retardation) – children (3–6 years old)	*n* = 1 unexposed, disease free	0/35 1/44	0.41 [0.02;10.40]	-
Severe cognitive developmental delay (Mental retardation) – children (> 6 years old)	*n* = 1 unexposed, WWE	21/5073 139/21,634	0.73 [0.46; 1.16]	-
Learning disorders	*n* = 1 unexposed, WWE; *n* = 1 unexposed, disease free	5^a^/125 4^a^/224	1.45 [0.82;2.54]	0%; 0

Bold indicates statistically significant results

*LTG* Lamotrigine, *ADHD* Attention deficit / hyperactivity disorder, *ASD* Autism spectrum disorder, *OR* Odds ratio, *95%CI* Confidence interval, *I²* Higgins heterogeneity test, *WWE* Women With Epilepsy

^a^Missing data

**Fig. 2:**
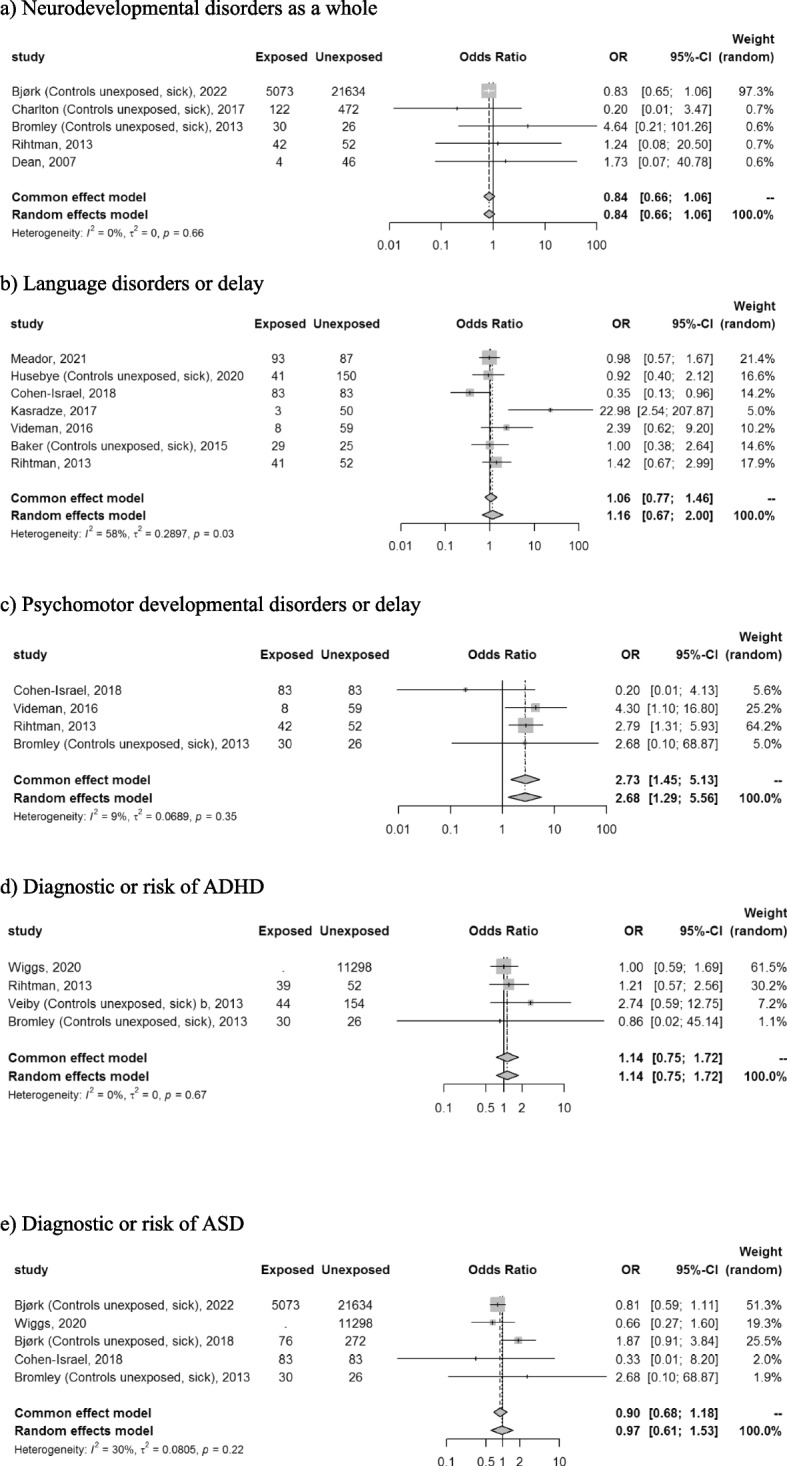
Forest plot of the risk of (**a**) Neuro-developmental disorders as a whole; (**b**) Language disorders or delay; (**c**) Psychomotor developmental disorders or delay; (**d**) Diagnosis or risk of attention deficit / hyperactivity disorder; (**e**) Diagnosis or risk of autism spectrum disorder

The risk of neurodevelopmental disorders considered as a whole by authors (i.e. including several cognitive and behavioral disorders as a whole) was reported in five studies [3, 12, 34, 37, 39]. The MA did not show a statistically significant association (pooled OR 0.84, 95% CI [0.66;1.06]; I² = 0%; tau²=0; *n* = 5,271 and 22,230 exposed and unexposed children respectively; Fig. 2a). The trim and fill method reported a very similar OR (Supplement S5a). A sensitivity analysis on the type of study could not be performed as all studies were cohorts (*n* = 5). The majority of the included studies used unexposed women with epilepsy as comparator (*n* = 4) and did not perform any adjustment (*n* = 4) (Supplement S6a).

The risk of language disorders or delay was assessed in seven studies [13–17, 31, 37]. There was no statistically significant increase in the occurrence of language disorders or delay (pooled OR 1.16, 95% CI [0.67;2.00]; I²=58%; tau²=0.29; *n* = 313 and 506 exposed and unexposed children respectively; Fig. 2b). After trim and fill, the pooled OR was similar (Supplement S5b). In the sensitivity analyses, when the type of control was restricted to untreated epileptic women, the results were consistent with the pooled OR (0.96, 95% [CI 0.51;1.80]; k = 2; I²=0) of the main analysis. The sensitivity analysis on the type of study could not be performed (*n* = 7 cohorts). Only one study performed an adjustment, but two of the remaining six studies performed a matching (Supplement S6b).

Four studies [13, 17, 37, 39] reported the risk for psychomotor developmental disorders or delay for which the MA found a statistically significant association (pooled OR 2.68, 95% CI [1.29;5.56]; I²=9%; tau²=0.07; *n* = 163 and 220 exposed and unexposed children respectively; Fig. 2c). Publication bias could not be assessed with Egger’s test and the trim and fill method was not applied because the funnel plot appeared symmetrical, meaning no publication bias was detected (Supplement S5c). The sensitivity analyses according to adjustment and type of studies could not be performed as all studies were homogenous. Although no results were adjusted, one study did use matching. In the sensitivity analysis on the type of comparator, a loss of statistical significance was observed in the sub-analysis using the comparator “unexposed, women with epilepsy” (*n* = 1 study, pooled OR 2.68, 95% CI [0.10;68.88]; I²=NA; Supplement S6c).

The diagnosis or risk of ADHD was reported in four studies [18, 37–39]. The pooled OR did not reach statistical significance (pooled OR 1.14, 95% CI [0.75;1.72]; I²=0%; tau²=0; n = at least 113 and 11,530 exposed and unexposed children respectively; Fig. 2d). The funnel plot did not lead to a trim and fill; no publication bias was detected (Supplement S5d). The sensitivity analysis according to the type of studies could not be performed. A majority of the included studies used unexposed women with epilepsy as comparator (*n* = 3) and no adjustment (*n* = 3); the pooled ORs were similar to that of the main analysis (Supplement S6d).

Five studies [3, 11, 13, 18, 39] addressed the diagnosis or risk of ASD. The MA showed no statistically significant association (pooled OR 0.97, 95% CI [0.61;1.53]; I²=30%; tau²=0.08; n = at least 5262 and 33,313 exposed and unexposed children respectively; Fig. 2e). No publication bias was highlighted by the funnel plot (Supplement S5e.). All included studies were cohorts meaning this parameter was not contributive to the sensitivity analysis. The majority of the included studies used unexposed women with epilepsy as comparator (*n* = 4); the pooled OR remained similar to that of the main analysis. Two studies performed an adjustment and among the three studies that did not, one performed a matching (Supplement S6e). Lastly, the pooled OR remained similar to that of the main after exclusion of the 2 studies with a partial overlapping with Bjørk et al. 2022 (Wiggs et al. 2020 and Bjørk et al. 2018): pooled OR 0.81, 95% CI [0.59;1.11] (I2 = 0% *n* = 5,186 exposed; 21,743 unexposed children).

The risk-of-bias was assessed using the ROBINS-I tool at an outcome-level. Overall, the confounding bias was frequently rated as critical/serious or unclear and other types of bias were considered low or moderate in at least 50% of the included studies (Supplement S7).

### Meta-analyses of neurodevelopmental disorders reported by less than four studies

An overview of the MA results for each outcome reported by less than four studies are presented in Table 2.

The cognitive developmental delay in infants under 3 years of age was investigated in two studies [17, 32]. In the MA including a total of 42 exposed infants, a statistically significant increase was obtained (pooled OR 3.42, 95% CI [1.17;9.98]; I²=0%; tau²=0; *n* = 42 and 86 exposed and unexposed children respectively). In older children, the risk was not statistically increased according to the MA carried out on 79 *in utero* exposed children aged 3–6 years old in three studies [15, 33, 37] (pooled OR 3.39, 95% CI [0.56;20.57]; I²=68%; tau²=1.70; *n* = 79 and 146 exposed and unexposed children respectively) and on 113 *in utero* exposed children older than 6 years of age in three studies [13, 31, 36] (pooled OR 0.75, 95% CI [0.41;1.37]; I²=0%; tau²=0; *n* = 113 and 124 exposed and unexposed children respectively). Two studies [3, 33] specifically evaluated severe cognitive developmental delay in 3–6 years old children and more than 6 years old children. For both, no statistically significant increase was obtained.

Learning disorders were assessed by two studies [13, 35]. A total of 125 *in utero* exposed children were evaluated, and the pooled OR was not statistically significant (pooled OR 1.45, 95% CI [0.82;2.54]; I²=0%; tau²=0; *n* = 125 and 224 exposed and unexposed children respectively).

## Discussion

The present systematic review and MA mainly assessed 5 outcomes investigated by at least 4 studies. Prenatal exposure to lamotrigine monotherapy in children born to women with epilepsy is not found to be statistically associated with neurodevelopmental disorders as a whole, language disorders or delay, diagnosis or risk of ASD and diagnosis or risk of ADHD, with a number of exposures ranging from 113 to 5271, depending on the outcome. On the contrary, the MA found that psychomotor developmental disorders were significantly associated with *in utero* exposure to lamotrigine monotherapy used for epilepsy. Cognitive developmental disorders and learning disorders have also been assessed but by fewer studies, and the MA reported a statistically significant increase of cognitive developmental disorders in prenatally lamotrigine-exposed infants younger than 3 years old but no significant association in the older age groups.

### Psychomotor developmental disorders or delay

The present MA retrieved a statistically significant association between psychomotor developmental disorders or delay and prenatal exposure to lamotrigine. To our knowledge, no other MA in the literature has reported this association. The 2017 network MA [2] reported a non-statistically significant pooled OR lower than the estimate herein (1.86 95%CI [0.72;4.76]; I² unknown; k unknown). Although the risk estimates remained similar in the sensitivity analyses according to the type of control group performed herein, the associations did not remain statistically significant, likely due to a loss of statistical power.

Interestingly, among the two studies who reported a significantly increased OR for psychomotor developmental disorders or delay, one study [37] showed only low or moderate risk for every type of bias assessed. The other study [17] was the only one to assess the risk of psychomotor developmental disorders or delay in very young infants (7 months old *versus* at least 4 years old in the other studies). This is intriguing as the sensitivity for detecting psychomotor developmental disorders or delay is known to increase with age [41]. As a child grows, he/she develops more complex skills and disorders and delays may thus appear in areas for which they were previously in line with their peers [16]. The study by Videman et al. [17] may thus reveal the most severe cases that can occur at younger ages. Nevertheless, a longer follow-up of the 7 months old infants might have allowed the authors to report a more constituted and accurate diagnosis [16]. Finally, it is important to note that only 163 *in utero* exposed children were assessed overall in the four included studies, further underlying the need for additional investigations to be carried out in order to clarify the association between psychomotor developmental disorders or delay and prenatal exposure to lamotrigine.

### Cognitive developmental delay

A total of 42 *in utero* exposed children were assessed in the two studies included in the MA. A cognitive developmental delay in infants younger than 3 years old was found to be significantly increased in lamotrigine-exposed pregnancies compared to controls. However, no significant association was found in the older age groups. A similar finding was reported in a study by Meador et al. [42], in which mean cognitive scores were rated by assessors using the differential ability scales (DAS) in children followed between the ages of 2, 3, 4, 5, and 6 years: Intelligence quotient (IQ) measures of children exposed to lamotrigine appeared to improve over time. When parents are informed of a cognitive delay diagnosis in their young child, they can be referred to intervention programs [42]. Eventually, children will then catch up and reach their age-standards as they grow, which is conceivable if the delay was not too severe. Unfortunately, the present analysis cannot corroborate whether the less than 3 years old children were severely delayed. Nevertheless, two studies [3, 33] did not report a statistically increased risk of severe delay in older children. The possibility of an incidental finding cannot be ruled out given that only 42 *in utero* exposed children less than 3 years old were evaluated.

### Autism spectrum disorder

In a previously published network MA [2], lamotrigine was associated with an increased occurrence of autism (traits and diagnosis combined) but no other neurodevelopmental defect. The same conclusion was not observed herein perhaps because of the methodological differences between the two MA protocols, notably the inclusion of polytherapies and the exclusion of outcomes reported as continuous variables in the MA published by Veroniki et al. in 2017 [2]. Moreover, since the release of this network MA, a large number of new studies investigating ASD were published [3, 11, 13, 18] and included in the MA herein (four out of the five studies included in the present MA were published after the publication of the network MA [2]).

### Strengths and limitations

Overall, the interpretation of the results was limited by the biases highlighted with the ROBINS-I tool. Confounding factors were not usually given enough consideration even though numerous prenatal, perinatal, and postnatal characteristics are known risk factors for neurodevelopmental disorders such as underlying genetic component, parental IQ, prematurity, socio-economic status, maternal age, etc. [31, 43–45]. These were either partially or not at all considered and may have thus impacted the findings. Furthermore, some countries don’t provide a basic healthcare system and that can generate disparities between countries in their ability to detect specific neurodevelopmental outcomes. This was taken into account by downgrading the risk-of-bias rating for selection bias and bias in outcome measurement.

Moreover, because no antiseizure medication has sufficient data regarding safety on neurodevelopmental outcomes after prenatal exposure, we chose to not consider an active comparator, excluding studies that compare antiseizure medications with each other. Nevertheless, the results obtained for lamotrigine should be considered in light with results for other antiseizure medications.

Because of the large number of scales and questionnaires measuring neurodevelopmental outcomes reported in the literature, we chose to build a neurodevelopmental correspondence matrix by aggregating different measurement tools. This was done in collaboration with psychiatrists (LJ and MN) to ensure clinical relevance. If the measurement tools considered for a single type of neurodevelopmental outcome had been too heterogeneous, this would have invalidated any clinical interpretation by oversimplifying the data and not taking into account possible subtleties. Conversely, if the correspondence matrix had been too discriminating, it would not have been possible to aggregate enough data. Nevertheless, the combination of data obtained in children of different ages and/or with different scales could introduce heterogeneity that should be discussed if necessary. Importantly, the developed matrix was found to be reliable and valuable for the classification of disorders assessed by tests in the present study. The use of the matrix was also a strength as it allowed to discard measurement tools that were identified as too weak or incomplete to fully assess the neurodevelopmental outcome of interest. This means that assessments obtained with tools deemed inadequate or insufficient were not included in the MA, thus avoiding dilution of the evidence collected with less reliable information.

Another strength of this MA was the exclusion of studies assessing lamotrigine for the treatment of other indications than epilepsy (e.g., bipolar disorders). This clinical homogeneity may have contributed to better capture baseline risks associated with neurodevelopment, therefore limiting confounding factors. Since the present work aimed to assess the potential functional deficits triggered by lamotrigine, only monotherapy interventions were included. This allowed the MA results to not be biased by the potential or proven neurodevelopmental impact of other ASM.

Neurodevelopmental outcomes are rarely addressed in the literature [1] partly because of the extensive follow-up needed. This leads to small sample sizes in studies that limit the ability to detect associations. Therefore, using an MA approach was a strength to study neurodevelopmental outcomes. In addition, the MA herein is currently the most up-to-date and therefore provides the latest state of the art in this field. Finally, the outcomes reported by at least four studies were not subject to publication bias according to the funnel plots.

### Implication for clinical practice

In case of pregnancy in a woman with epilepsy, and preferably when a pregnancy is planned, the benefit-risk of an ASM treatment must be re-evaluated. Although lamotrigine is increasingly prescribed [4, 46], the present MA underlines the fact that there is still limited evidence regarding its impact on neurodevelopmental outcomes in children prenatally exposed to lamotrigine monotherapy. Larger studies assessing children with long-term follow-up are required to closely monitor neurodevelopmental outcomes. Given the high use of lamotrigine and the emerging evidence regarding its impact on neurodevelopmental outcomes, the results of the present MA call for special attention to be directed towards these children.

## Conclusion

Prenatal exposure to lamotrigine monotherapy in children born to women with epilepsy is not found to be statistically associated with neurodevelopmental disorders as a whole, language disorders or delay, diagnosis or risk of ASD and diagnosis or risk of ADHD. On the contrary, the MA found an increased risk of psychomotor developmental disorders or delay and cognitive developmental delay in less than 3 years old children (but not in the older age groups) after *in utero* exposure to lamotrigine monotherapy. Nevertheless, these findings were based exclusively on observational studies presenting biases and were limited by the small number of children included. There is still limited evidence regarding the impact of prenatal exposure to lamotrigine on neurodevelopmental outcomes. More studies should assess the risk of neurodevelopmental outcomes in children prenatally exposed to lamotrigine.

### Supplementary Information

**Additional file 1.**

## Data Availability

The datasets generated and/or analysed during the current study are available in the metaPreg repository at metapreg.org, and are available from the corresponding author on reasonable request.

## Abbreviations

ADHD: Attention Deficit / Hyperactivity Disorder
ASD: Autism Spectrum Disorder
ASM: Antiseizure Medications
BMI: Body Mass Index
DAS: Differential Ability Scales
IQ: Intelligence Quotient
MA: Meta-analysis
NOS: Not Otherwise Specified
OR: Odd Ratio
WWE: Women With Epilepsy
