# Supplementary material for: Neurodevelopmental outcomes after prenatal exposure to lamotrigine monotherapy in women with epilepsy: a systematic review and meta-analysis
Source: BMC Pregnancy Childbirth. 2024 Feb 2;24:103. doi: 10.1186/s12884-023-06242-9 (PMC10835851; doi:10.1186/s12884-023-06242-9)
Supplement: Supplementary file 1 — Additional file 1. [file 12884_2023_6242_MOESM1_ESM.docx]

**Supplementary Material**

Supplement S1: Search request for Medline electronic database.

Supplement S2: Neurodevelopmental correspondence matrix.

Supplement S3: Data collection form.

Supplement S4: Management of study overlaps.

Supplement S5: Funnel plot assessing publication bias.

Supplement S6: Sensitivity analyses.

Supplement S7: Risk-of-bias assessment at an outcome-level using the ROBINS-I tool.

Supplement S8: MOOSE checklist.

Supplement S1: Search request for Medline electronic database.

(“Anticonvulsants”) AND ("2015"[Date - Publication] : "3000"[Date - Publication]) AND ("birth defects" OR "birth defects-drug exposure" OR "teratogenic risk" OR "teratogenicity" OR "prenatal exposure" OR "prenatally exposed" OR "fetal exposure" OR "congenital anomaly" OR "fetal anomalies" OR "congenital anomalies" OR "congenital malformation" OR "congenital malformations" OR "congenital major malformations" OR "congenital disorders" OR "cardiovascular defects" OR "preterm birth" OR "stillbirth" OR "miscarriage " OR "spontaneous abortion" OR "use during pregnancy" OR "exposure in pregnancy" OR "exposure during pregnancy" OR "exposed in utero" OR "first-trimester exposure" OR Teratogens OR "Birth defect"[Mesh] OR "Congenital Abnormalities"[Mesh] OR "Fetal Death/chemically induced"[Mesh] OR "Fetal Development/drug effects"[Mesh] OR "Fetal Diseases/chemically induced"[Mesh] OR "Fetus/drug effects"[Mesh] OR "Stillbirth"[Mesh] OR "Teratogens"[Mesh] OR "Abortion, Spontaneous"[Mesh]) AND ((“Autistic Disorder”[Mesh] OR “Autism Spectrum Disorder”[Mesh] OR “Neurodevelopmental Disorders”[Mesh]) AND pregnancy) AND ("cohort study" OR "prospective study" OR "prospective observational study" OR "case-control study" OR "prospective follow-up study" OR "prospective follow-up" OR "meta-analysis" OR "systematic review" OR "retrospective study" OR "registry" OR "birth register" OR "observational study" OR "population-based health datasets" OR "population health data" OR "Cohort Studies"[Mesh] OR "Prospective Studies"[Mesh] OR "Case-Control Studies"[Mesh] OR "Observational Study"[ptyp] OR "Meta-Analysis"[ptyp] OR "Longitudinal Studies"[Mesh] OR "Registries"[Mesh] OR "Retrospective Studies"[Mesh] OR "Randomized Controlled Trial"[ptyp] OR "matched controls" OR "matched control" OR "case and control" OR "compared with controls" OR "case-control" OR "healthy controls" OR disproportionality OR "proportional reporting ratio" OR "odds ratio" OR "hazard ratio")

Supplement S2: Neurodevelopmental correspondence matrix.

| **Adverse outcome** | **Definitions of the criteria and measurement tools that can be used for this dimension** |
| --- | --- |
| **Neurodevelopmental disorders (as a whole)** | 1. Neurodevelopmental disorders as a whole, without specific diagnosis: several cognitive disorders (e.g. Cognitive delay, speech therapy, ...) **and** at least one behavioral disorder (ADHD, ASD, …)    - Medical records (**ICD-10** **F70-F90.9**)    - Additional/special educational needs… |
| **Cognitive developmental delay – infants**  (< 3 years old) | 1. Diagnosis of developmental delay in medical record: confirmed diagnosis of **moderate** cognitive developmental delay by developmental pediatrician or pediatric neurologist 2. Global DQ **≈< 85 or < 1 SD** assessed with:    - Griffiths mental development scales    - Bayley Scales of Infant Development (*corrected for number of weeks born prematurely*)    - Denver Developmental Screening Test (DDST)    - Wechsler Preschool and Primary Scale of Intelligence (WPPSI)    - Non-verbal intelligence test of Snijders-Oomen (SON-R)    - the Revised Amsterdam Children’s Intelligence Test (RAKIT)    - Early childhood development scale of Brunet-Lézine    - Mullen Scales of Early Learning 3. Performance IQ or Mental Development Index in global scales 4. Low overall score on an Adaptive Behavior scale (with homogeneity in the scale’s domains):    - VINELAND-II - Vineland adaptative behavior scale (or Vineland Social Maturity Scale – old name)    - ABAS II (Adaptive behavior assessment system; parents and/or teachers) |
| **Cognitive developmental delay – children** (3-6 years old) | 1. Diagnosis of developmental delay in medical record: confirmed diagnosis of **moderate** cognitive developmental delay by developmental pediatrician or pediatric neurologist 2. Global IQ **≈< 85 or < 1 SD** assessed with:    - Wechsler Intelligence Scale for Children (WISC)    - Wechsler Preschool and Primary Scale of Intelligence (WPPSI)    - Differential Ability Scales (DAS)    - Non-verbal intelligence test of Snijders-Oomen (SON-R)    - the Revised Amsterdam Children’s Intelligence Test (RAKIT)    - Color Progressive Matrices (CPM or PM47)    - Color Progressive Matrices (CPM-BF)    - Standard Progressive Matrices (SPM or PM38)    - Stanford-Binet intelligence scales    - McCarthy Scales of Children’s Abilities 3. Performance IQ or Mental Development Index in global scales 4. Low overall score on an Adaptive Behavior scale (with homogeneity in the scale’s domains):    - VINELAND-II - Vineland adaptative behavior scale (or Vineland Social Maturity Scale – old name)    - ABAS II (Adaptive behavior assessment system; parents and/or teachers) |
| **Cognitive developmental delay – children** (> 6 years old) |  |
| **Severe cognitive developmental delay (Mental retardation) – infants**  (< 3 years old) | 1. Medical records (**ICD-10: F70-F79)** (**ICD-9: 317-319)** 2. Medical record: confirmed diagnosis of **severe** cognitive developmental delay by developmental pediatrician or pediatric neurologist 3. Global DQ **≈< 70 or < 2 SD** assessed with:    - Griffiths mental development scales    - Bayley Scales of Infant Development (*corrected for number of weeks born prematurely*)    - Denver Developmental Screening Test (DDST)    - Wechsler Preschool and Primary Scale of Intelligence (WPPSI)    - Non-verbal intelligence test of Snijders-Oomen (SON-R)    - the Revised Amsterdam Children’s Intelligence Test (RAKIT)    - Differential Ability Scales (DAS)    - Early childhood development scale of Brunet-Lézine 4. Low overall score on an Adaptive Behavior scale (with homogeneity in the scale’s domains):    - VINELAND-II - Vineland adaptative behavior scale (or Vineland Social Maturity Scale – old name)    - ABAS II (Adaptive behavior assessment system; parents and/or teachers)) 5. Severe learning disorders (school results…), with indication of global cognitive delay |
| **Severe cognitive developmental delay (Mental retardation) – children**  (3-6 years old) | 1. Medical records (**ICD-10: F70-F79)** (**ICD-9: 317-319)** 2. Medical record: confirmed diagnosis of **severe** cognitive developmental delay by developmental pediatrician or pediatric neurologist 3. Global IQ **≈< 70 or < 2 SD** assessed with:    - Griffiths mental development scales    - Bayley Scales of Infant Development    - Denver Developmental Screening Test (DDST)    - Wechsler Preschool and Primary Scale of Intelligence (WPPSI) before 5 years old    - Wechsler Intelligence Scale for Children (WISC) after 5 years old    - Wechsler Adult Intelligence Scale (WAIS) after 16 years old    - Non-verbal intelligence test of Snijders-Oomen (SON-R)    - the Revised Amsterdam Children’s Intelligence Test (RAKIT)    - Differential ability scales (DAS) 4. Low overall score on an Adaptive Behavior scale (with homogeneity in the scale’s domains):    - VINELAND-II - Vineland adaptative behavior scale (or Vineland Social Maturity Scale – old name)    - ABAS II (Adaptive behavior assessment system; parents and/or teachers) 5. Severe learning disorders (school results…), with indication of global cognitive delay |
| **Severe cognitive developmental delay (Mental retardation) – children**  (> 6 years old) |  |
| **Language disorders or delay**  (all ages) | 1. Medical record (**ICD-10: F80)**: Confirmed diagnosis of language delay by developmental pediatrician or pediatric neurologist 2. Dysphasia 3. Verbal IQ **< 85** or **< 1 SD**, or language delay assessed with:    - the Reynell Developmental Language Scale    - Clinical Evaluation of Language Fundamentals (CELF)    - Learning Accomplishment Profile    - “Hearing and Language” subset in Griffiths scales    - Verbal subset of IQ or DQ    - Communication subset VINELAND    - Communication and Symbolic Behavior Scales Developmental Profile (CSBS-DP)    - Comprehensive Language Assessment    - the Phelps Kindergarten Readiness Scale II (*if no other test assesses language*)    - Peabody Picture Vocabulary Test; Receptive Expressive Emergent Language Scale; Expressive One Word Picture Vocabulary Test; or Sequenced Inventory of Communication Development, … 4. Medical record: Need for speech therapy |
| **Learning disorders**  (all ages) | 1. Medical record (**ICD-10: F81; DSM:315 except 315.4)**: Confirmed diagnosis of learning disorder by developmental pediatrician or pediatric neurologist 2. Dyslexia, dysorthographia, … (*except* *dysphasia and dyspraxia*) 3. Academic performances (spelling, reading, math, …) |
| **Psychomotor developmental disorders or delay**  (all ages) | 1. Medical record: Diagnosis of psychomotor delay recorded in medical records, diagnosis of neuromotor deficit confirmed by a trained nurse practitioner; infant failing to sit by 10 months of age or walk by 18 months of age… 2. Dyspraxia (**ICD-10: F82; DSM: 315.4)** 3. Psychomotor IQ **≈< 85 or < 1 SD** assessed with:    - Miller Function and Participation Scales (M-FUN)    - Psychomotor subsets:      - Griffiths scales      - BSID II (Bayley Scales of Infant Development, motor scale)      - Schedule of Growing Skills II (SGS II; Locomotion = gross motor; Handling = Fine motor)      - Motor VINELAND    - Touwen’s test    - The Alberta Infant Motor Scale (AIMS) |
| **Risk of autism spectrum disorder (ASD)**  (all ages) | 1. Risk of ASD **(< 18 months old)**, assessed with:    - Checklist for Autism in Toddlers (CHAT)    - Modified Checklist Autism for Toddlers (M-CHAT)    - Modified Checklist Autism for Toddlers Revised with Follow- up (M-CHAT-R/F)    - Quantitative Checklist for Autism (Q-CHAT) 2. Risk of ASD **(≥ 18 months old)**, assessed with:    - The 40-item Social Communication Questionnaire (SCQ)    - Modified Autism Spectrum Screening Questionnaire (ASSQ)    - Autism Screening Questionnaire (ASQ; ASQ-II)    - Autism-spectrum Quotient (AQ)    - Childhood Autism Rating Scale (CARS) when score between 27-29    - Social Responsiveness Scale (SRS)    - Social Emotional Questionnaire |
| **Diagnosis of autism spectrum disorder (ASD)**  (all ages) | 1. ASD or subtype of ASD (Childhood autism, Atypical autism, Rett syndrome, Other childhood disintegrative disorder, Overactive disorder associated with mental retardation and stereotyped movements, Asperger syndrome, Other pervasive developmental disorders or unspecified): 2. Medical records (**ICD-9: 299**) (**ICD-10: F84.0-F84.9)** 3. Medical records (**DSM-IV or DSM-V**) 4. Diagnosis of ASD (global) assessed with:    - Childhood Autism Rating Scale (CARS) when score >30    - Autism Diagnostic Observation Schedule (ADOS)    - Revised Behavior Summarized Evaluation scale (BSE-R)    - Autism Diagnostic Interview (ADI)    - Autism Diagnostic Interview – revised (ADI-R) |
| **Diagnosis of autism spectrum disorder (ASD) or risk (when diagnosis unavailable)** | See the categories of ASD above. |
| **Risk of attention deficit hyperactivity disorder** (**ADHD)**  (all ages) | 1. Risk of ADHD, whatever the age, but assessed by **one investigator with**:    - Conners’ rating scales (parents or teachers)    - Attention Problems and Hyperactivity Scales e.g. Strengths and Difficulties Questionnaire (SDQ); Behavior assessment system for children (BASC; parents or teachers)    - Child Behavior Checklist (CBCL)    - Social Emotional Questionnaire |
| **Diagnosis of attention deficit hyperactivity disorder (ADHD)**  (all ages) | 1. Diagnosis of ADHD reported in medical record: DSM-IV or DSM-V or ICD (**ICD10: F90**) or confirmed diagnosis in hospital/medical records made by a pediatrician or child psychiatrist 2. Treatment for ADHD (methylphenidate, …) 3. ADHD, assessed by **several investigators (≥2)**    - Conners’ rating scales (parents and teachers)    - Attention Problems and Hyperactivity Scales e.g. Strengths and Difficulties Questionnaire (SDQ); Behavior assessment system for children (BASC; parents and teachers)    - Child Behavior Checklist (CBCL) |
| **Diagnosis of attention deficit hyperactivity disorder (ADHD) or risk (when diagnosis unavailable)** | See the categories of ADHD above. |

Supplement S3: Data collection form.

| Study | Wording | Outcome | Extra-cted OR | ll | ul | X1 | N1 | X0 | N0 | Exposition period | Control type |
| --- | --- | --- | --- | --- | --- | --- | --- | --- | --- | --- | --- |
| Baker, 2015 | Mean child Full scale IQ - Differential ability scales (DAS) (age mean >6 years old) | Cognitive developmental delay - children (> 6 years old) | 1.84 | 0.91 | 3.72 | NA | 29 | NA | 210 | during pregnancy (anytime or not specified) | unexposed, disease free |
| Baker, 2015 | Mean child verbal IQ - Differential ability scales (DAS) (age mean >6 years old) | Language disorders/delay | 1.82 | 0.90 | 3.68 | NA | 29 | NA | 210 | during pregnancy (anytime or not specified) | unexposed, disease free |
| Baker, 2015 | Mean child Full scale IQ - Differential ability scales (DAS) (age mean >6 years old) | Cognitive developmental delay - children (> 6 years old) | 1.16 | 0.44 | 3.07 | NA | 29 | NA | 25 | during pregnancy (anytime or not specified) | unexposed, sick |
| Baker, 2015 | Mean child verbal IQ - Differential ability scales (DAS) (age mean >6 years old) | Language disorders/delay | 1.00 | 0.38 | 2.64 | NA | 29 | NA | 25 | during pregnancy (anytime or not specified) | unexposed, sick |
| Bjørk, 2018 | Autistic traits at 18 months and/or at 36 months (M-CHAT <2/6 critical or <3/23 or SCQ >13) | Risk of ASD (Autism spectrum disorder) | NA | NA | NA | 13 | 76 | 6907 | 75497 | during pregnancy (anytime or not specified) | unexposed, disease free |
| Bjørk, 2018 | Autistic traits at 18 months and/or at 36 months (M-CHAT <2/6 critical or <3/23 or SCQ >13) | Risk of ASD (Autism spectrum disorder) | NA | NA | NA | 13 | 76 | 27 | 272 | during pregnancy (anytime or not specified) | unexposed, sick |
| Bjørk, 2022 | Neurodevelopmental Disorder (ND): any diagnoses of autism spectrum disorder (F84.0, F84.1, F84.5) or intellectual disability (F70, F71, F72, F73) plus F84.3, F84.4, F84.8, F84.9, F79 (between 6.1 and 7.9 years old) | Neuro-developmental disorders (as a whole) | NA | NA | NA | 81 | 5073 | 68295 | 4463879 | during pregnancy (anytime or not specified) | unexposed (general population or NOS) |
| Bjørk, 2022 | Diagnosis of childhood autism (F84.0), atypical autism (F84.1), and Asperger syndrome (F84.5) (between 6.1 and 7.9 years old) | Diagnosis of ASD (Autism spectrum disorder) | NA | NA | NA | 49 | 5073 | 38437 | 4463879 | during pregnancy (anytime or not specified) | unexposed (general population or NOS) |
| Bjørk, 2022 | Diagnosis of intellectual disability (ID): mild ID (F70), moderate ID (F71), severe ID (F72), and profound ID (F73) (between 6.1 and 7.9 years old) | Severe cognitive developmental delay (Mental retardation) - children (> 6 years old) | NA | NA | NA | 21 | 5073 | 16384 | 4463879 | during pregnancy (anytime or not specified) | unexposed (general population or NOS) |
| Bjørk, 2022 | Neurodevelopmental Disorder (ND): any diagnoses of autism spectrum disorder (F84.0, F84.1, F84.5) or intellectual disability (F70, F71, F72, F73) plus F84.3, F84.4, F84.8, F84.9, F79 (between 6.1 and 7.9 years old) | Neuro-developmental disorders (as a whole) | 0.83 | 0.65 | 1.06 | 81 | 5073 | 443 | 21634 | during pregnancy (anytime or not specified) | unexposed, sick |
| Bjørk, 2022 | Diagnosis of childhood autism (F84.0), atypical autism (F84.1), and Asperger syndrome (F84.5) (between 6.1 and 7.9 years old) | Diagnosis of ASD (Autism spectrum disorder) | 0.81 | 0.59 | 1.11 | 49 | 5073 | 267 | 21634 | during pregnancy (anytime or not specified) | unexposed, sick |
| Bjørk, 2022 | Diagnosis of intellectual disability (ID): mild ID (F70), moderate ID (F71), severe ID (F72), and profound ID (F73) (between 6.1 and 7.9 years old) | Severe cognitive developmental delay (Mental retardation) - children (> 6 years old) | 0.73 | 0.46 | 1.16 | 21 | 5073 | 139 | 21634 | during pregnancy (anytime or not specified) | unexposed, sick |
| Bromley, 2010 | Overall ability below average performance (≤ 84) (Griffiths) (mean age 10 months old) | Cognitive developmental delay - infants (< 3 years old) | NA | NA | NA | 5 | 34 | 18 | 230 | during pregnancy (anytime or not specified) | unexposed, disease free |
| Bromley, 2013 | Dyspraxia (at 6 years old) | Psychomotor developmental disorders/delay | NA | NA | NA | 1 | 30 | 0 | 214 | during pregnancy (anytime or not specified) | unexposed, disease free |
| Bromley, 2013 | Neurodevelopmental disorder (at 6 years old) | Neuro-developmental disorders (as a whole) | 4.06 | 0.55 | 22.20 | 2 | 30 | 4 | 214 | during pregnancy (anytime or not specified) | unexposed, disease free |
| Bromley, 2013 | Autistic Spectrum Disorders (at 6 years old) | Diagnosis of ASD (Autism spectrum disorder) | NA | NA | NA | 1 | 30 | 4 | 214 | during pregnancy (anytime or not specified) | unexposed, disease free |
| Bromley, 2013 | Attention deficit hyperactivity disorder (at 6 years old) | Diagnosis of ADHD (Attention deficit hyperactivity disorder) | NA | NA | NA | 0 | 30 | 0 | 214 | during pregnancy (anytime or not specified) | unexposed, disease free |
| Bromley, 2010 | Overall ability below average performance (≤ 84) (Griffiths) (mean age 10 months old) | Cognitive developmental delay - infants (< 3 years old) | NA | NA | NA | 5 | 34 | 2 | 27 | during pregnancy (anytime or not specified) | unexposed, sick |
| Bromley, 2013 | Dyspraxia (at 6 years old) | Psychomotor developmental disorders/delay | NA | NA | NA | 1 | 30 | 0 | 26 | during pregnancy (anytime or not specified) | unexposed, sick |
| Bromley, 2013 | Neurodevelopmental disorder (at 6 years old) | Neuro-developmental disorders (as a whole) | NA | NA | NA | 2 | 30 | 0 | 26 | during pregnancy (anytime or not specified) | unexposed, sick |
| Bromley, 2013 | Autistic Spectrum Disorders (at 6 years old) | Diagnosis of ASD (Autism spectrum disorder) | NA | NA | NA | 1 | 30 | 0 | 26 | during pregnancy (anytime or not specified) | unexposed, sick |
| Bromley, 2013 | Attention deficit hyperactivity disorder (at 6 years old) | Diagnosis of ADHD (Attention deficit hyperactivity disorder) | NA | NA | NA | 0 | 30 | 0 | 26 | during pregnancy (anytime or not specified) | unexposed, sick |
| Charlton, 2017 | Neurodevelopmental disorders (at 6 years old) | Neuro-developmental disorders (as a whole) | NA | NA | NA | 0 | 122 | 58 | 6048 | during pregnancy (anytime or not specified) | unexposed, disease free |
| Charlton, 2017 | Neurodevelopmental disorders (at 6 years old) | Neuro-developmental disorders (as a whole) | NA | NA | NA | 0 | 122 | 9 | 472 | during pregnancy (anytime or not specified) | unexposed, sick |
| Cohen-Israel, 2018 | Autism (a majority between 6-12 years old) | Diagnosis of ASD (Autism spectrum disorder) | NA | NA | NA | 0 | 83 | 1 | 83 | 1st trimester | unexposed, disease free |
| Cohen-Israel, 2018 | Developmental delay (a majority between 6-12 years old) | Cognitive developmental delay - children (> 6 years old) | NA | NA | NA | 12 | 83 | 19 | 83 | 1st trimester | unexposed, disease free |
| Cohen-Israel, 2018 | Speech delay (a majority between 6-12 years old) | Language disorders/delay | NA | NA | NA | 6 | 83 | 15 | 83 | 1st trimester | unexposed, disease free |
| Cohen-Israel, 2018 | Motor delay (a majority between 6-12 years old) | Psychomotor developmental disorders/delay | NA | NA | NA | 0 | 83 | 2 | 83 | 1st trimester | unexposed, disease free |
| Cohen-Israel, 2018 | Learning disabilities (a majority between 6-12 years old) | Learning disorders | NA | NA | NA | 5 | 83 | 4 | 83 | 1st trimester | unexposed, disease free |
| Cummings, 2011 | Mild or significant delay (score ≥1, <2 SD or ≥2 SD below the mean) (Bayley Scales of Infant Development or the Griffiths Scale) (mean age in years: m1=2.7 and m0=4.3) | Cognitive developmental delay - children (3-6 years old) | 1.10 | 0.10 | 13.70 | 1 | 35 | 2 | 44 | throughout pregnancy | unexposed, disease free |
| Cummings, 2011 | Significant delay (score ≥2 SD below the mean) (Bayley Scales of Infant Development or the Griffiths Scale) (mean age in years: m1=2.7 and m0=4.3) | Severe cognitive developmental delay (Mental retardation) - children (3-6 years old) | NA | NA | NA | 0 | 35 | 1 | 44 | throughout pregnancy | unexposed, disease free |
| Dean, 2007 | Neurodevelopmental disorder (mean age in years: m1=1.5 and m0=15.25) | Neuro-developmental disorders (as a whole) | NA | NA | NA | 0 | 4 | 3 | 46 | during pregnancy (anytime or not specified) | unexposed, sick |
| Elkjaer, 2018 | Danish at 6th grade (mean age 12.9 years old) | Learning disorders | 1.49 | 0.80 | 2.76 | NA | 42 | NA | 141 | during pregnancy (anytime or not specified) | unexposed, sick |
| Gopinath, 2015 | Full Scale IQ < 86 (Wechsler Intelligence Scale for Children (WISC-IV)) (at 10-12 years of age) | Cognitive developmental delay - children (> 6 years old) | NA | NA | NA | 0 | 1 | 11 | 16 | during pregnancy (anytime or not specified) | unexposed, sick |
| Husebye, 2018 | Global language delay 36 months (>1.5 SD below) the Ages and Stages Questionnaires (ASQ) | Language disorders/delay | NA | NA | NA | 5 | 57 | 3133 | 57367 | during pregnancy (anytime or not specified) | unexposed, disease free |
| Husebye, 2020 | Language impairment age 8 years (Language 20) | Language disorders/delay | 1.20 | 0.60 | 2.60 | 9 | 41 | 8250 | 42550 | during pregnancy (anytime or not specified) | unexposed, disease free |
| Husebye, 2018 | Global language delay 36 months (>1.5 SD below) the Ages and Stages Questionnaires (ASQ) | Language disorders/delay | NA | NA | NA | 5 | 57 | 12 | 204 | during pregnancy (anytime or not specified) | unexposed, sick |
| Husebye, 2020 | Language impairment age 8 years (Language 20) | Language disorders/delay | NA | NA | NA | 9 | 41 | 35 | 150 | during pregnancy (anytime or not specified) | unexposed, sick |
| Kasradze, 2017 | Full scale IQ - Wechsler Preschool and Primary Scale of Intelligence (WPPSI-4) (mean age 4-4.5 years old) | Cognitive developmental delay - children (3-6 years old) | 30.10 | 3.28 | 276.62 | NA | 3 | NA | 50 | during pregnancy (anytime or not specified) | unexposed, disease free |
| Kasradze, 2017 | Verbal comprehension (VCI) (WPPSI-4) (mean age 4-4.5 years old) | Language disorders/delay | 22.98 | 2.54 | 207.85 | NA | 3 | NA | 50 | during pregnancy (anytime or not specified) | unexposed, disease free |
| Meador, 2021 | Language Score in 2 years old children with the Bayley Scales of Infant and Toddler Development (BSID-III) | Language disorders/delay | 0.98 | 0.57 | 1.66 | NA | 93 | NA | 87 | 3rd trimester | unexposed, disease free |
| Rihtman, 2013 | Children: special education (mean age 4-5 years old) | Neuro-developmental disorders (as a whole) | NA | NA | NA | 1 | 42 | 1 | 52 | at least 1st trimester | unexposed (general population or NOS) |
| Rihtman, 2013 | Stanford Binet Intelligence Scales, Fifth Edition (SB5) - GIQ (mean age 4-5 years old) | Cognitive developmental delay - children (3-6 years old) | 1.65 | 0.78 | 3.49 | NA | 41 | NA | 52 | at least 1st trimester | unexposed (general population or NOS) |
| Rihtman, 2013 | Stanford Binet Intelligence Scales, Fifth Edition (SB5) - Verbal IQ (VIQ) (mean age 4-5 years old) | Language disorders/delay | 1.42 | 0.67 | 2.98 | NA | 41 | NA | 52 | at least 1st trimester | unexposed (general population or NOS) |
| Rihtman, 2013 | Miller Function and Participation Scales (MFUN) - Fine Motor (Preschool children: mean age 4-5y) | Psychomotor developmental disorders/delay | 2.79 | 1.31 | 5.92 | NA | 42 | NA | 52 | at least 1st trimester | unexposed (general population or NOS) |
| Rihtman, 2013 | Conners' Parent - ADHD Index (Preschool children: mean age 4-5y) | Risk of ADHD (Attention deficit hyperactivity disorder) | 1.21 | 0.57 | 2.56 | NA | 39 | NA | 52 | at least 1st trimester | unexposed (general population or NOS) |
| Veiby b, 2013 | ADHD symptoms ≥ 2SD (at 36 months) (Specific checklist) (Parent-reported) | Risk of ADHD (Attention deficit hyperactivity disorder) | 1.50 | 0.40 | 4.80 | 3 | 44 | 1743 | 43571 | during pregnancy (anytime or not specified) | unexposed, disease free |
| Veiby b, 2013 | ADHD symptoms ≥ 2SD (at 36 months) (Specific checklist) (Parent-reported) | Risk of ADHD (Attention deficit hyperactivity disorder) | NA | NA | NA | 3 | 44 | 4 | 154 | during pregnancy (anytime or not specified) | unexposed, sick |
| Videman, 2016 | Griffiths Mental Developmental Scale - General quotient (at the age of 7 months) | Cognitive developmental delay - infants (< 3 years old) | 4.57 | 1.17 | 17.90 | NA | 8 | NA | 59 | during pregnancy (anytime or not specified) | unexposed, disease free |
| Videman, 2016 | Griffiths Mental Developmental Scale - Hearing and speech (at the age of 7 months) | Language disorders/delay | 2.39 | 0.62 | 9.19 | NA | 8 | NA | 59 | during pregnancy (anytime or not specified) | unexposed, disease free |
| Videman, 2016 | Griffiths Mental Developmental Scale - Locomotor (at the age of 7 months) | Psychomotor developmental disorders/delay | 4.30 | 1.10 | 16.79 | NA | 8 | NA | 59 | during pregnancy (anytime or not specified) | unexposed, disease free |
| Wiggs, 2020 | Austism Spectrum Disorder (after 2 years of age) | Diagnosis of ASD (Autism spectrum disorder) | 0.66 | 0.27 | 1.58 | NA | NA | NA | 11298 | at least 1st trimester | unexposed, sick |
| Wiggs, 2020 | Attention-deficit/hyperactivity disorder (after 2 years of age) | Diagnosis of ADHD (Attention deficit hyperactivity disorder) | 1.00 | 0.59 | 1.69 | NA | NA | NA | 11298 | at least 1st trimester | unexposed, sick |

NA, not available; extracted.OR, odds ratio extracted; ll, inferior limit of the 95% confidence interval; ul, upper limit of the 95% confidence interval; X1, number of exposed cases; N1, total number of exposed; X0, number of unexposed cases; N0, total number of unexposed.

Supplement S4: Management of study overlaps.

| **The overlaps (Author, publication year)** | **Selected study** | **Justification** |
| --- | --- | --- |
| - Baker, 2015^16^ - Bromley, 2010^29^ | Baker, 2015 | Same database but language disorders or delay was assessed in older children. |
| - Bjørk, 2018^17^ - Veiby, 2013^24^ | Bjørk, 2018 | Same cohort but the diagnosis or risk of ASD was assessed in a larger population of *in utero* exposed children. |
| - Charlton, 2017^18^ - Baker, 2015^16^ - Bromley, 2013^3^ - Bromley, 2010^29^ - Bromley, 2008^30^ | Bromley, 2013 | Same database but the outcome of neuro-developmental disorders as a whole was better defined. The psychomotor developmental disorders or delay was evaluated in a larger population of *in utero* exposed children, older children, and using a better scale (diagnosis). The diagnosis or risk of ASD updated preliminary findings presented in Bromley et al., 2008. At last, Charlton et al., 2017 reported the same data for comparison purposes. |
| - Husebye, 2020^21^ - Husebye, 2018^39^ | Husebye, 2020 | Same cohort but the children assessed were older. |

Supplement S5: Funnel plot assessing publication bias with trim and fill (left) and asymmetry test p-value by Egger’s regression (right) for: a. Neuro-developmental disorders; b. Language disorders or delay; c. Psychomotor developmental disorders or delay; d. Diagnosis or risk of attention deficit hyperactivity disorder; e. Diagnosis or risk of autism spectrum disorder.


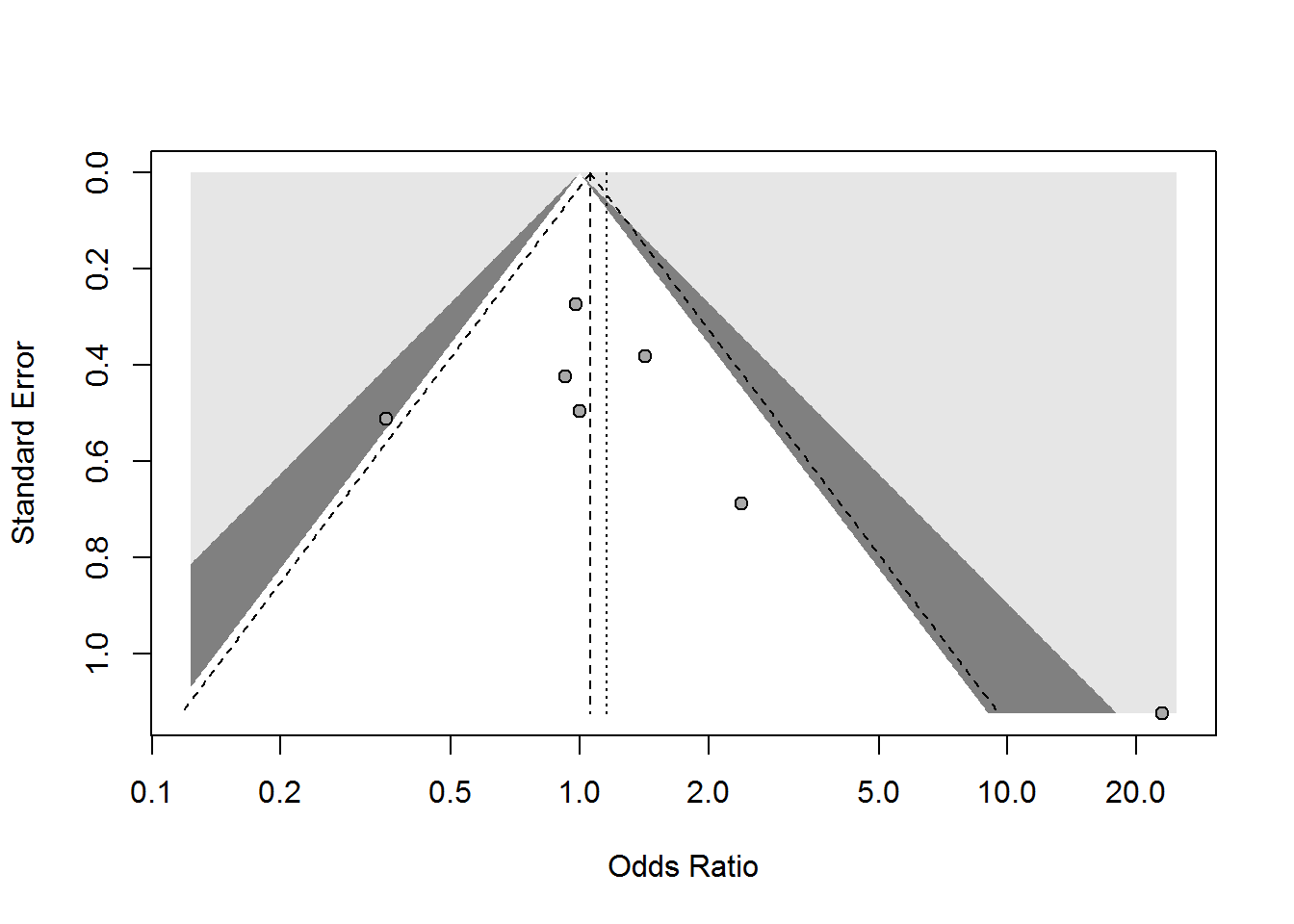

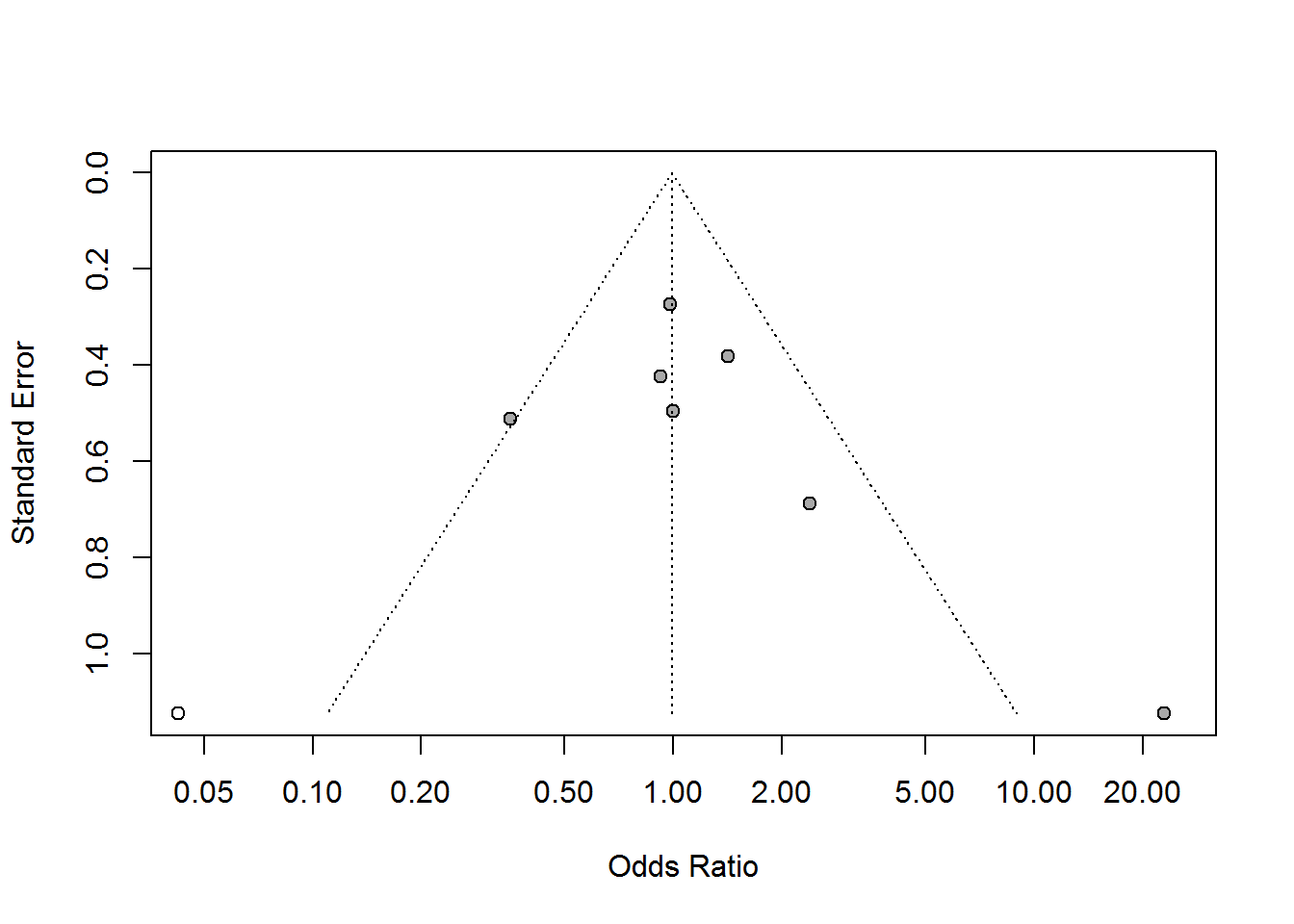

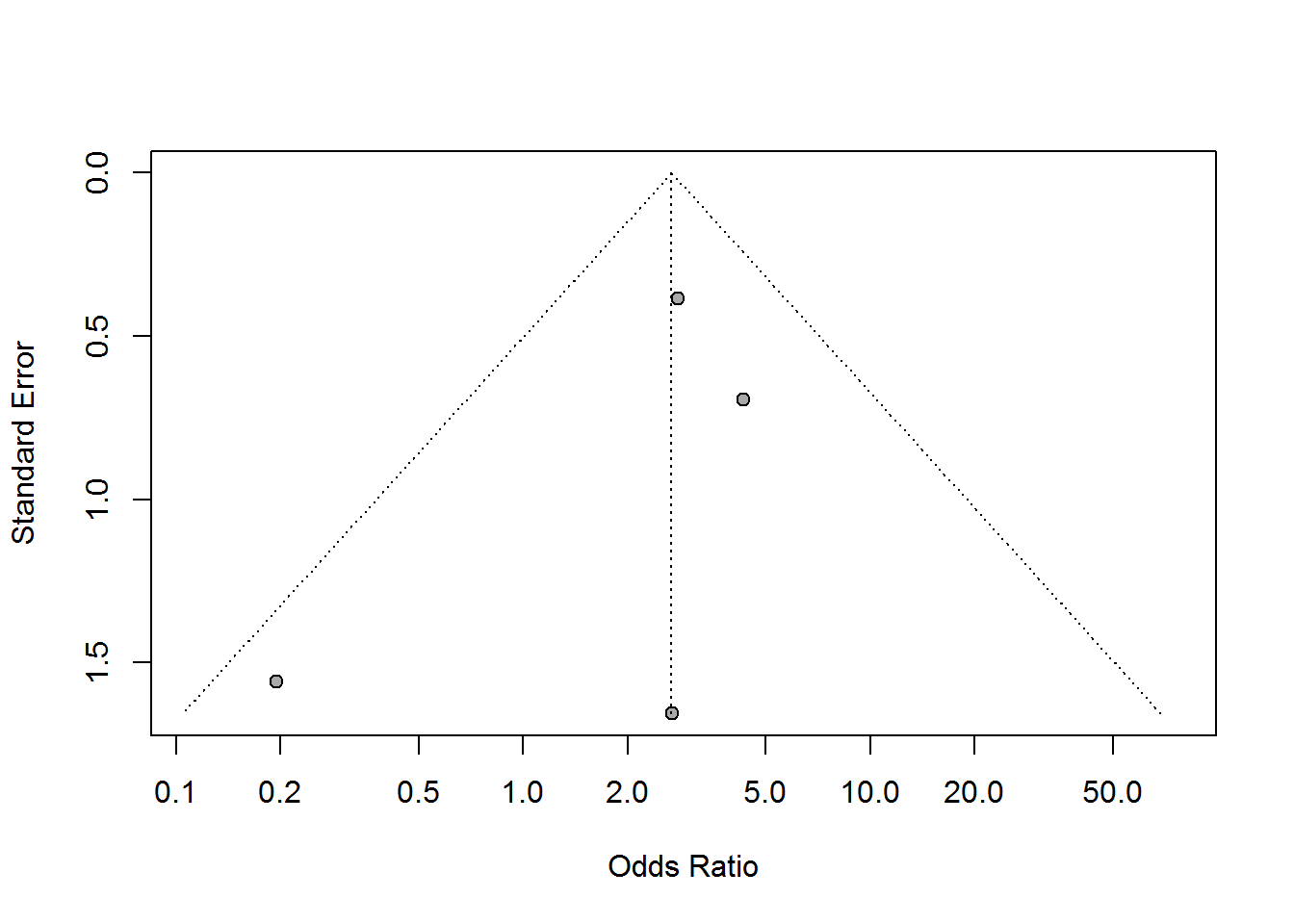

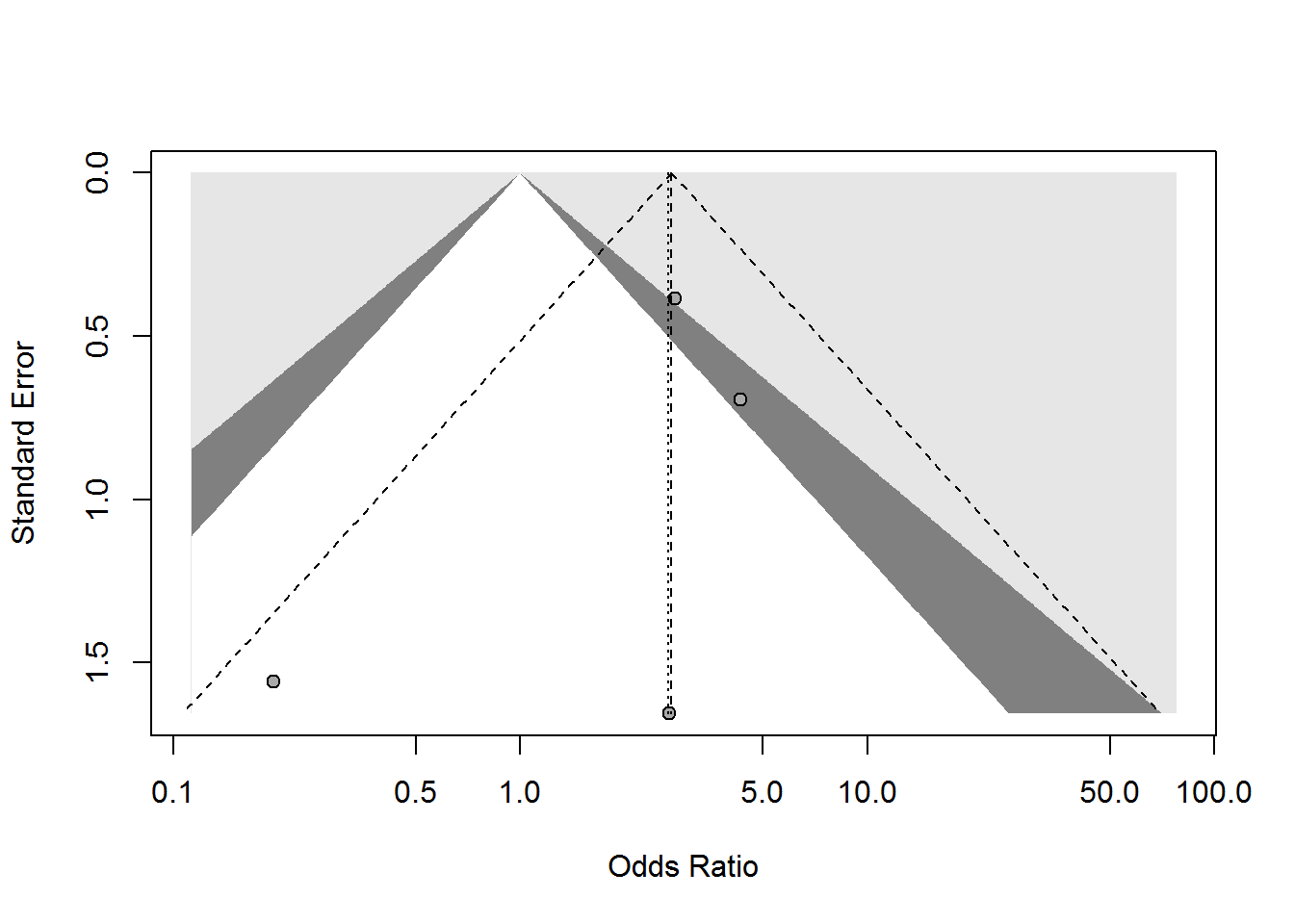


Asymmetry test p-value: not calculable

Asymmetry test p-value: not calculable

Asymmetry test p-value: not calculable

a.

b.


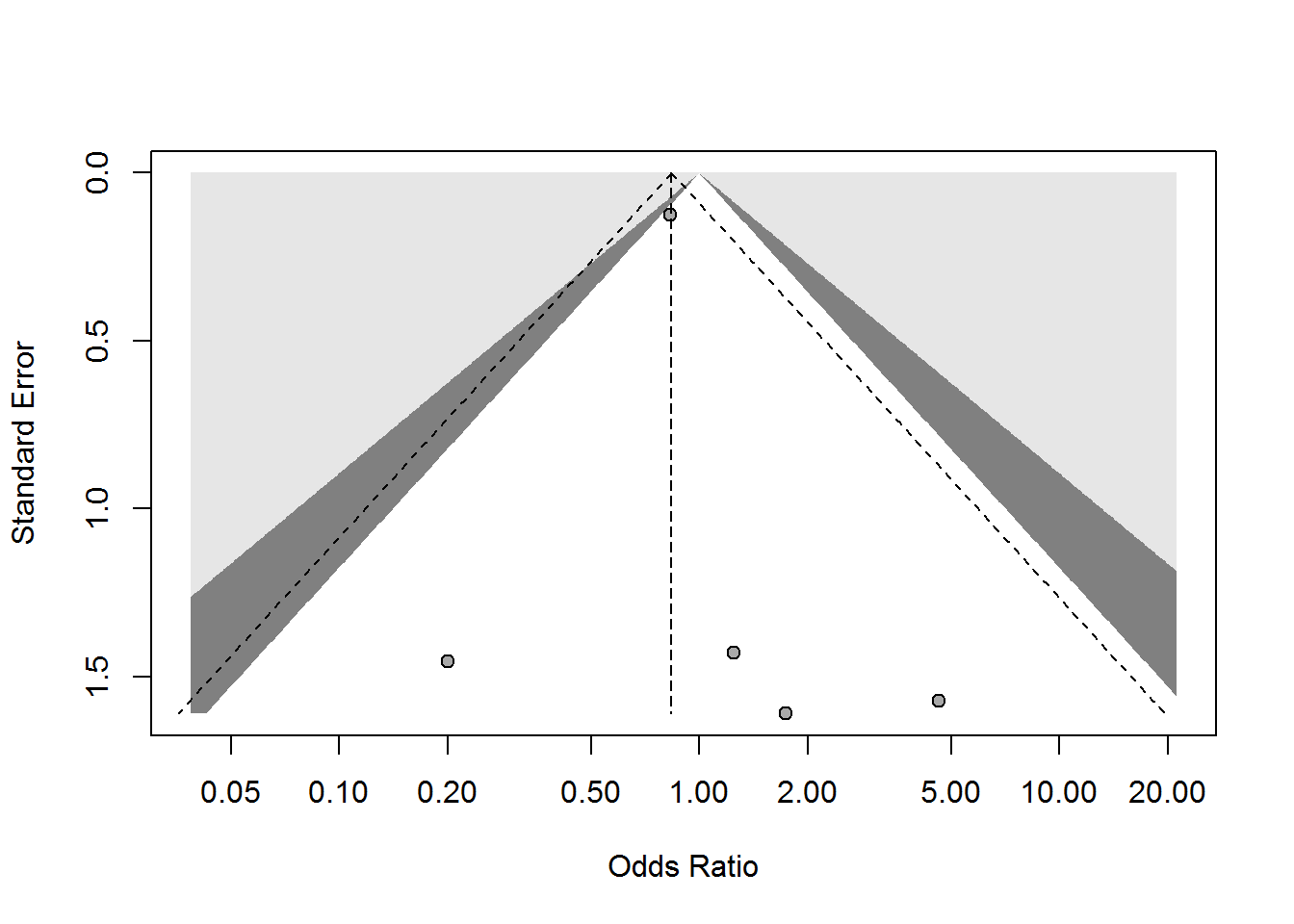

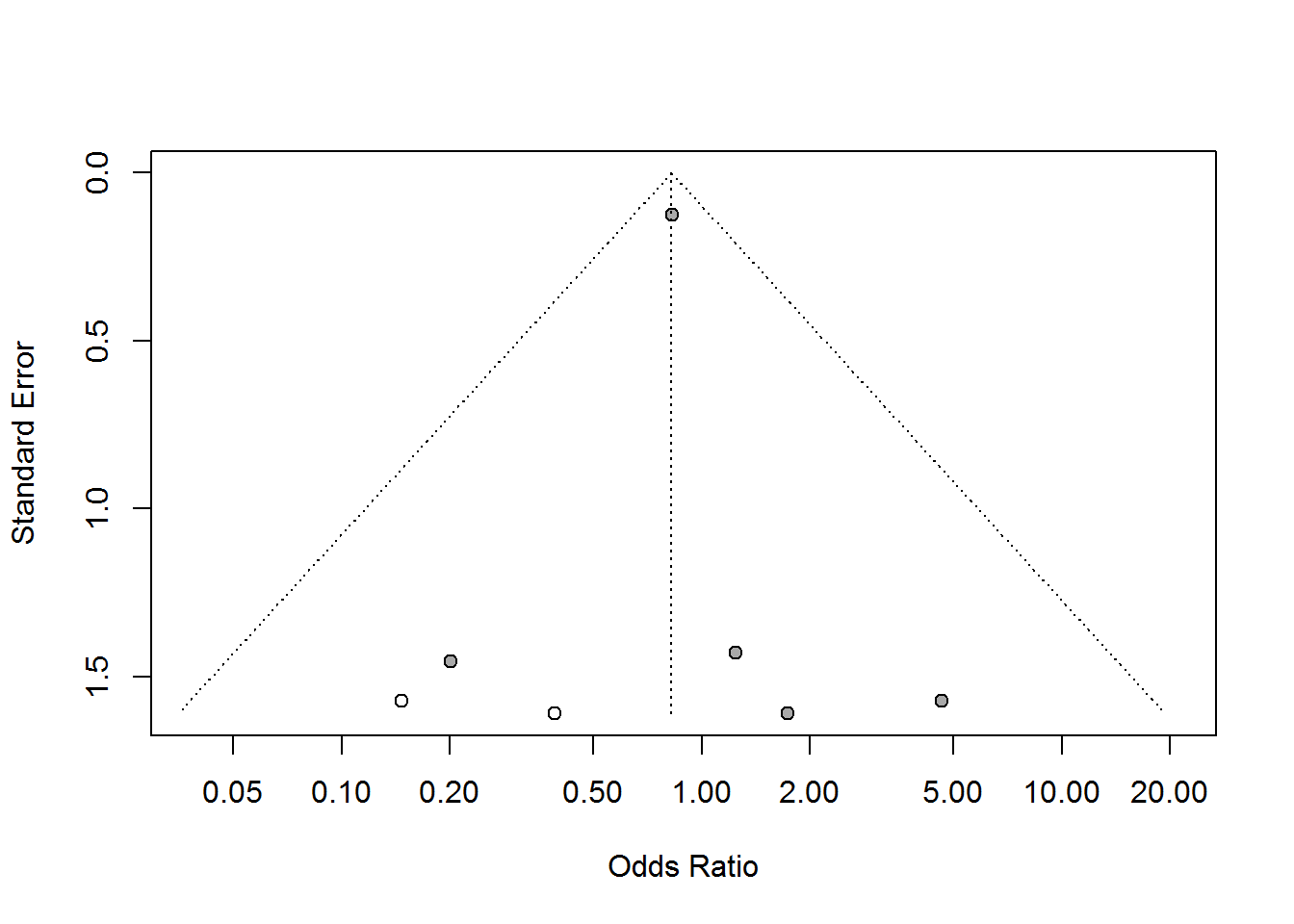


Trim and fill: n=1 added study

OR 0.99, 95%CI [0.52;1.88]

Trim and fill: n=0 added study

Trim and fill: n=2 added studies

OR 0.82, 95%CI [0.65;1.05]

# c.

Continue next page


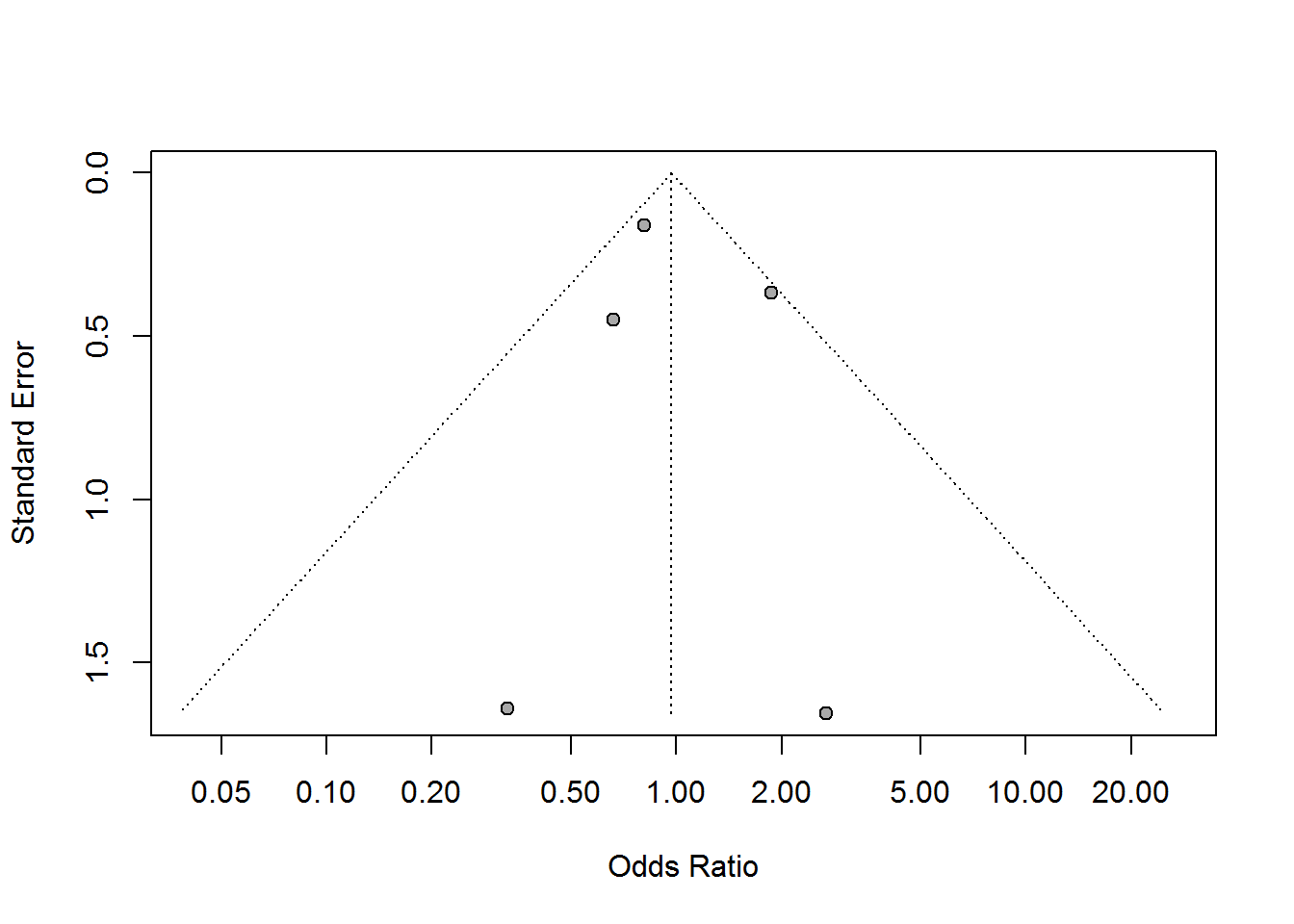

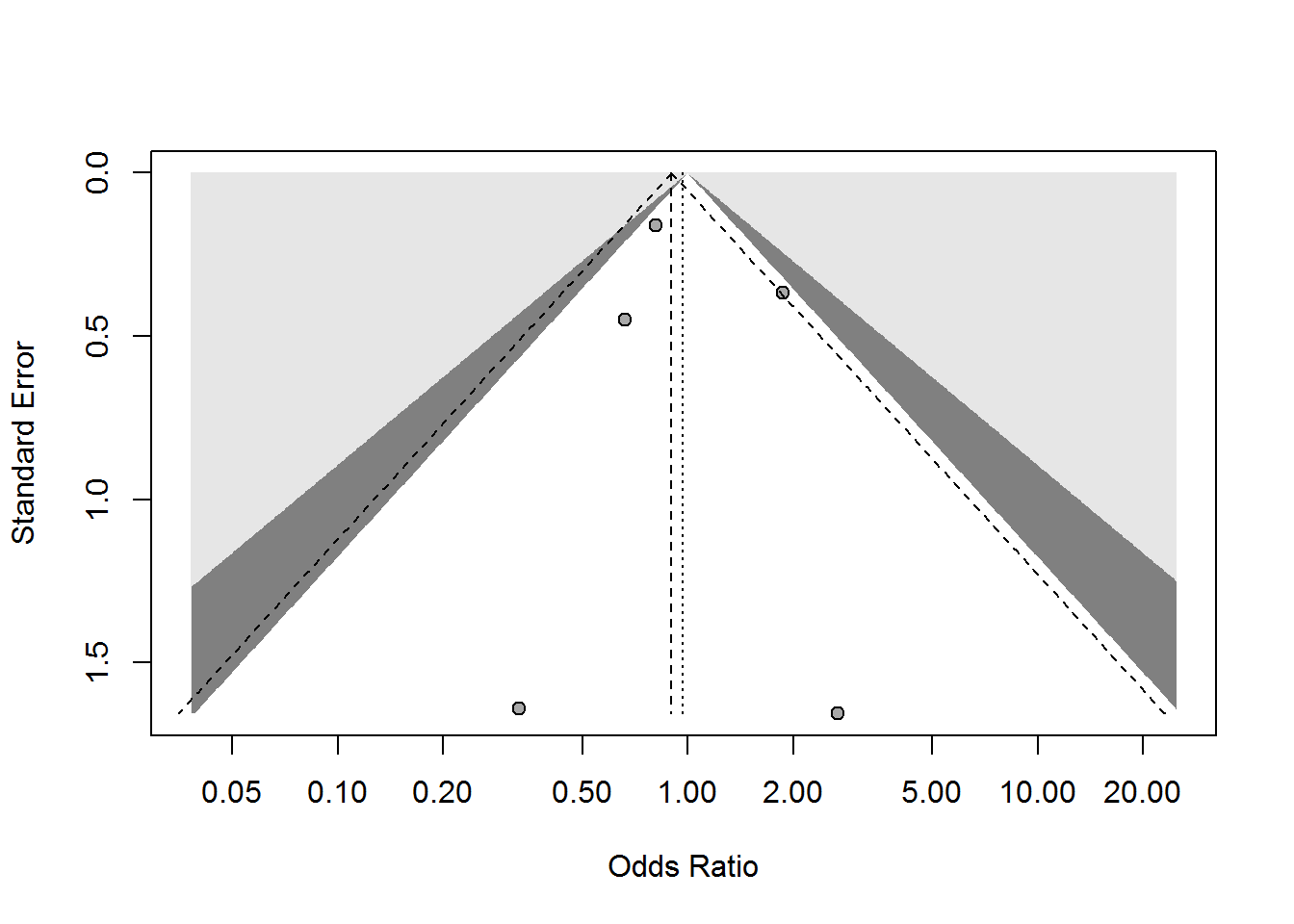

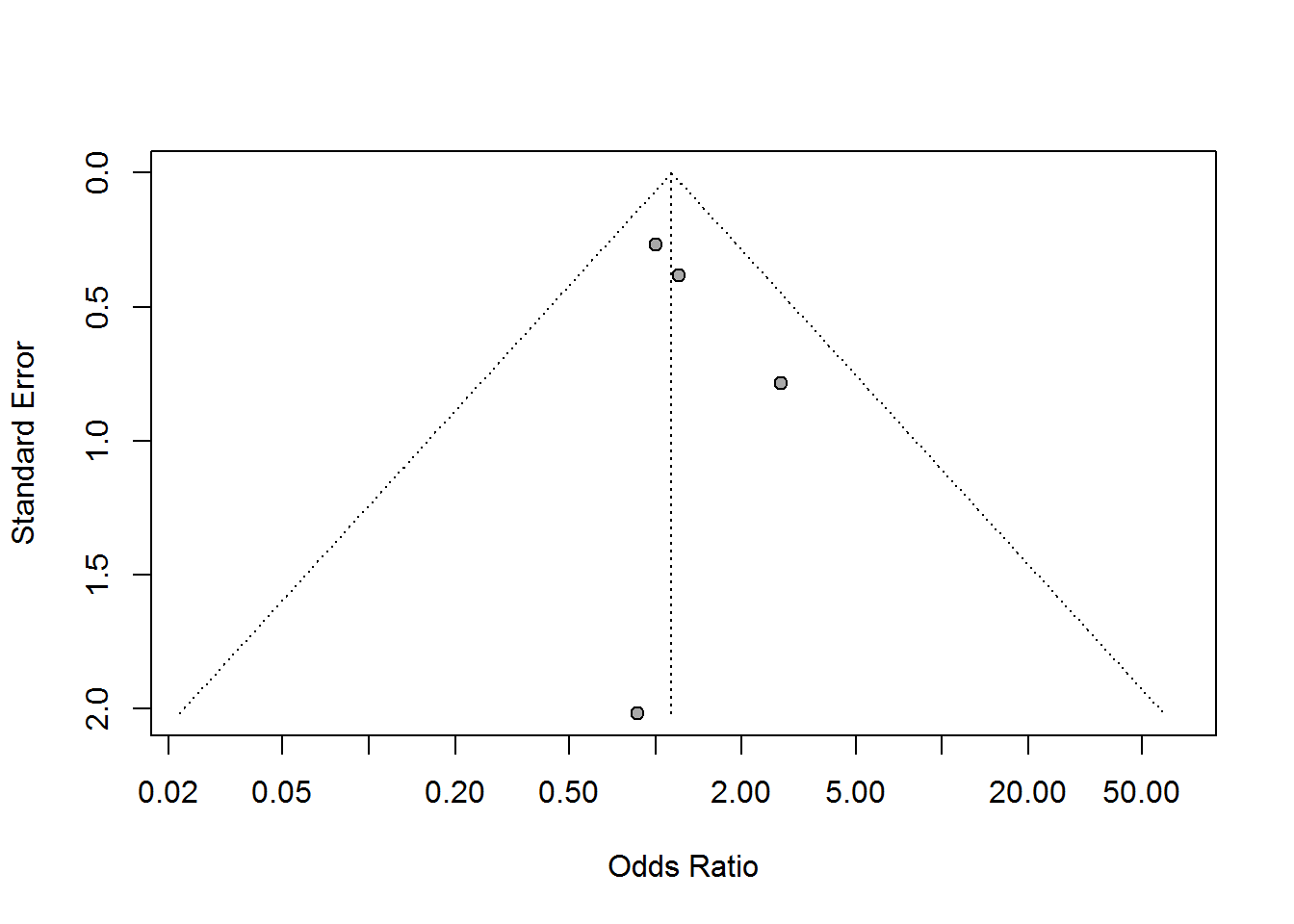

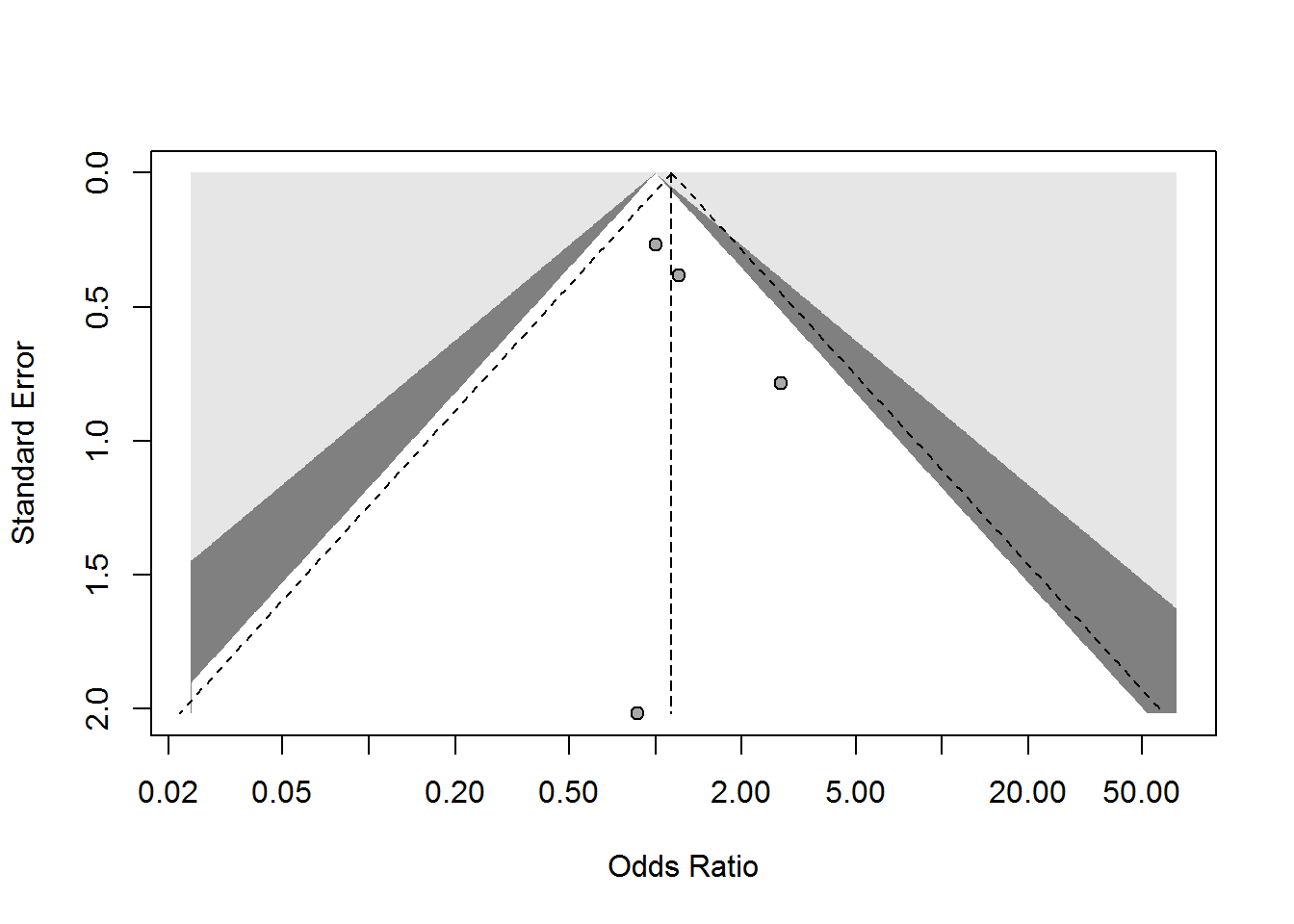


e.

Asymmetry test p-value: not calculable

# d.

Trim and fill: n=0 added study

Asymmetry test p-value: not calculable

Trim and fill: n=0 added study


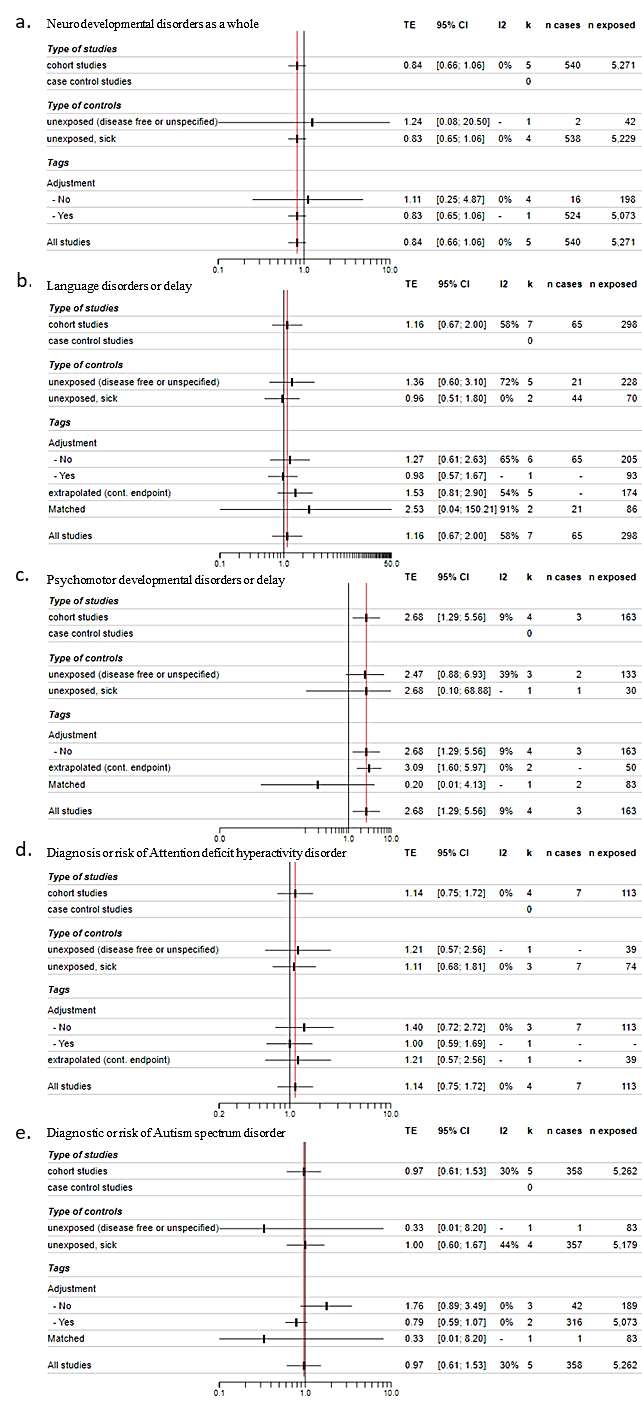
Supplement S6: Sensitivity analyses for: a. Neurodevelopmental disorders as a whole; b. Language disorders or delay; c. Psychomotor developmental disorders or delay; d. Diagnosis or risk of attention deficit hyperactivity disorder; e. Diagnosis or risk of autism spectrum disorder. TE, treatment effect; 95% CI, confidence interval; I², Higgins heterogeneity test; k, number of included studies; n cases, number of children with outcome, n exposed, number of lamotrigine exposures; extrapolated (cont. endpoint), number of included studies reporting continuous endpoint. Odds ratios are presented on a log scale.

Supplement S7: Risk-of-bias assessment at an outcome-level using the ROBINS-I tool.

Neuro-developmental disorders as a whole

Language disorders or delay

Psychomotor developmental disorders or delay

Diagnosis or risk of attention deficit hyperactivity disorder (ADHD)

Diagnosis or risk of autism spectrum disorder (ASD)


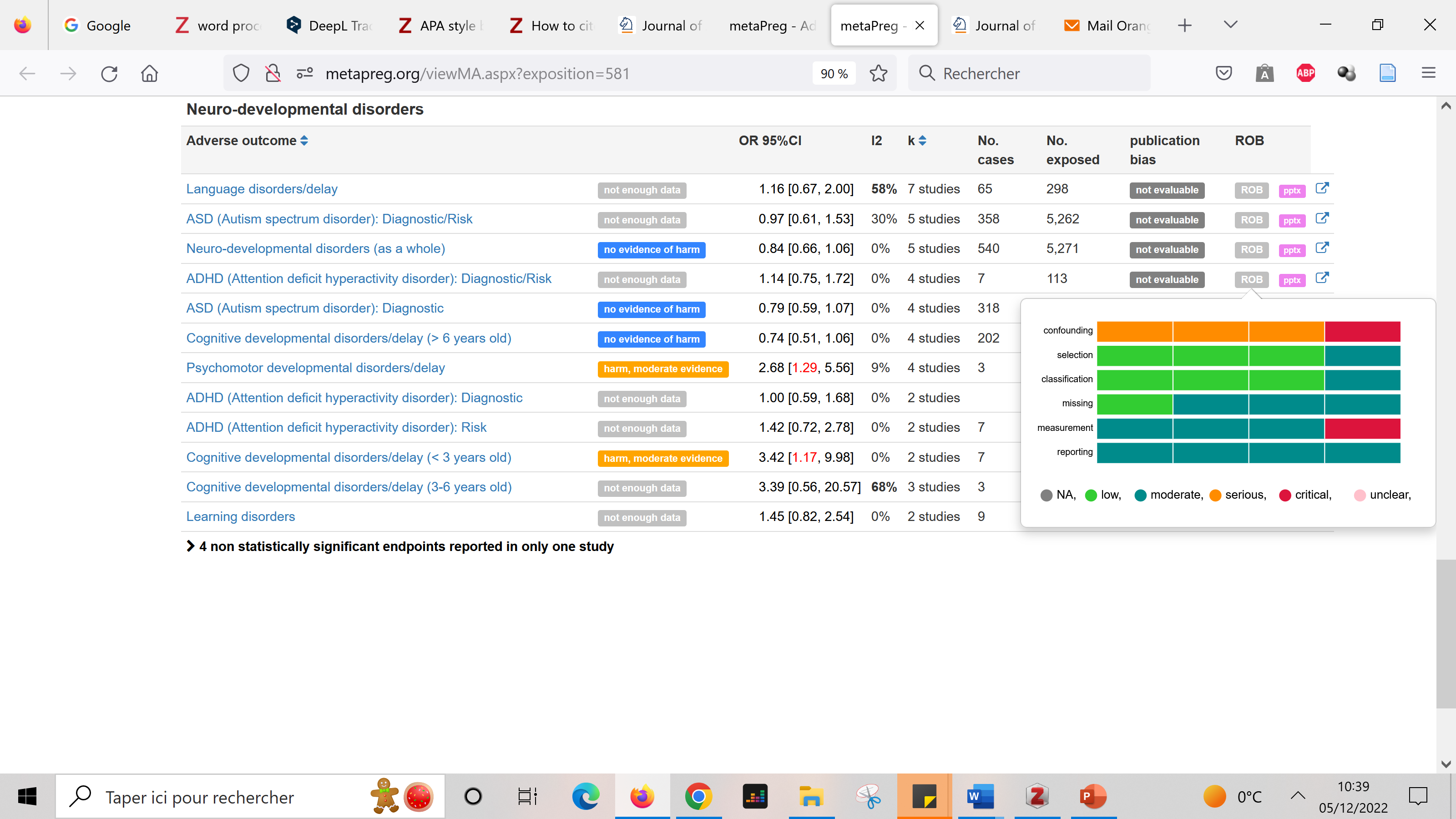

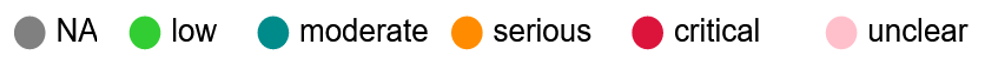

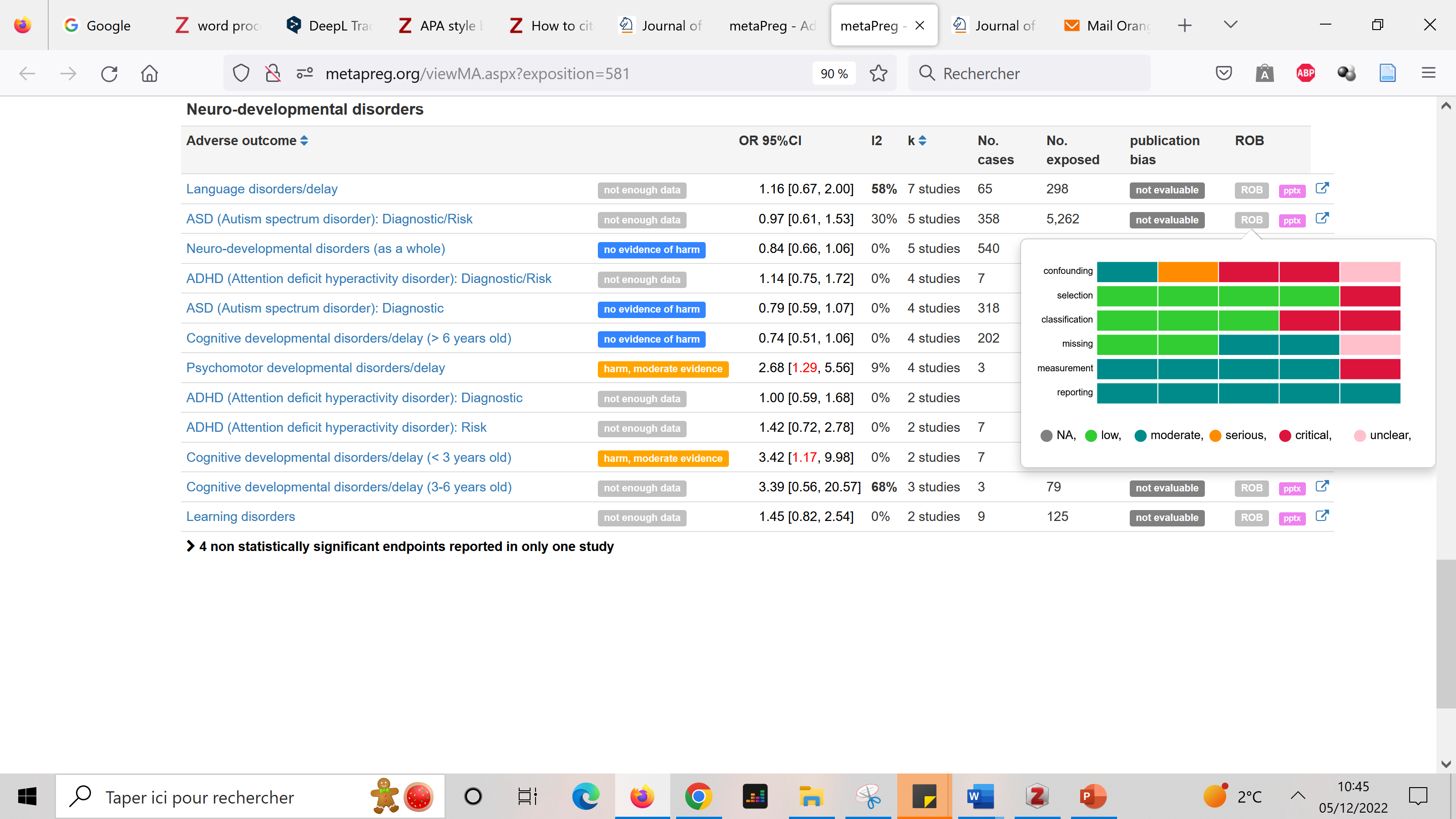

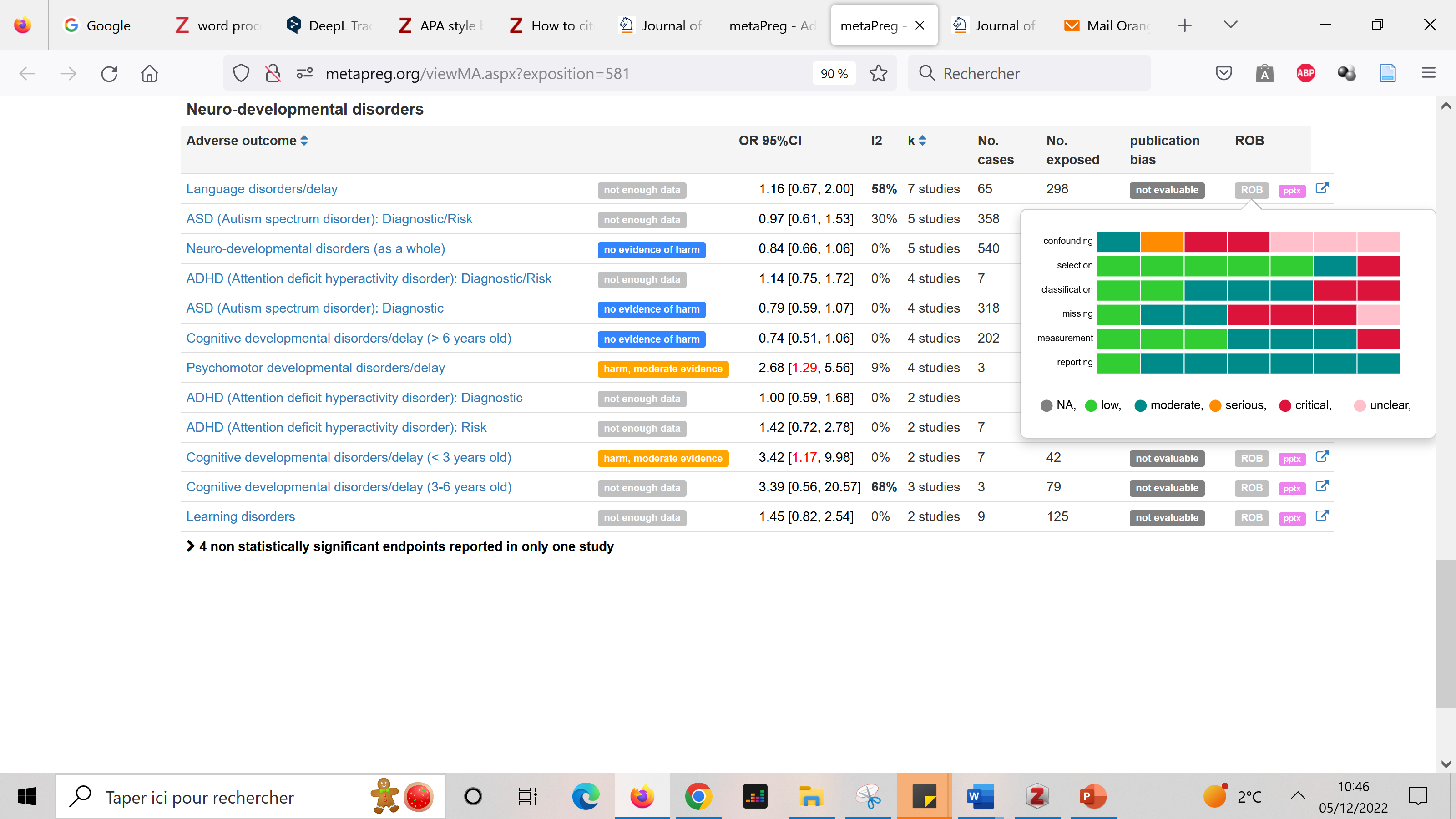

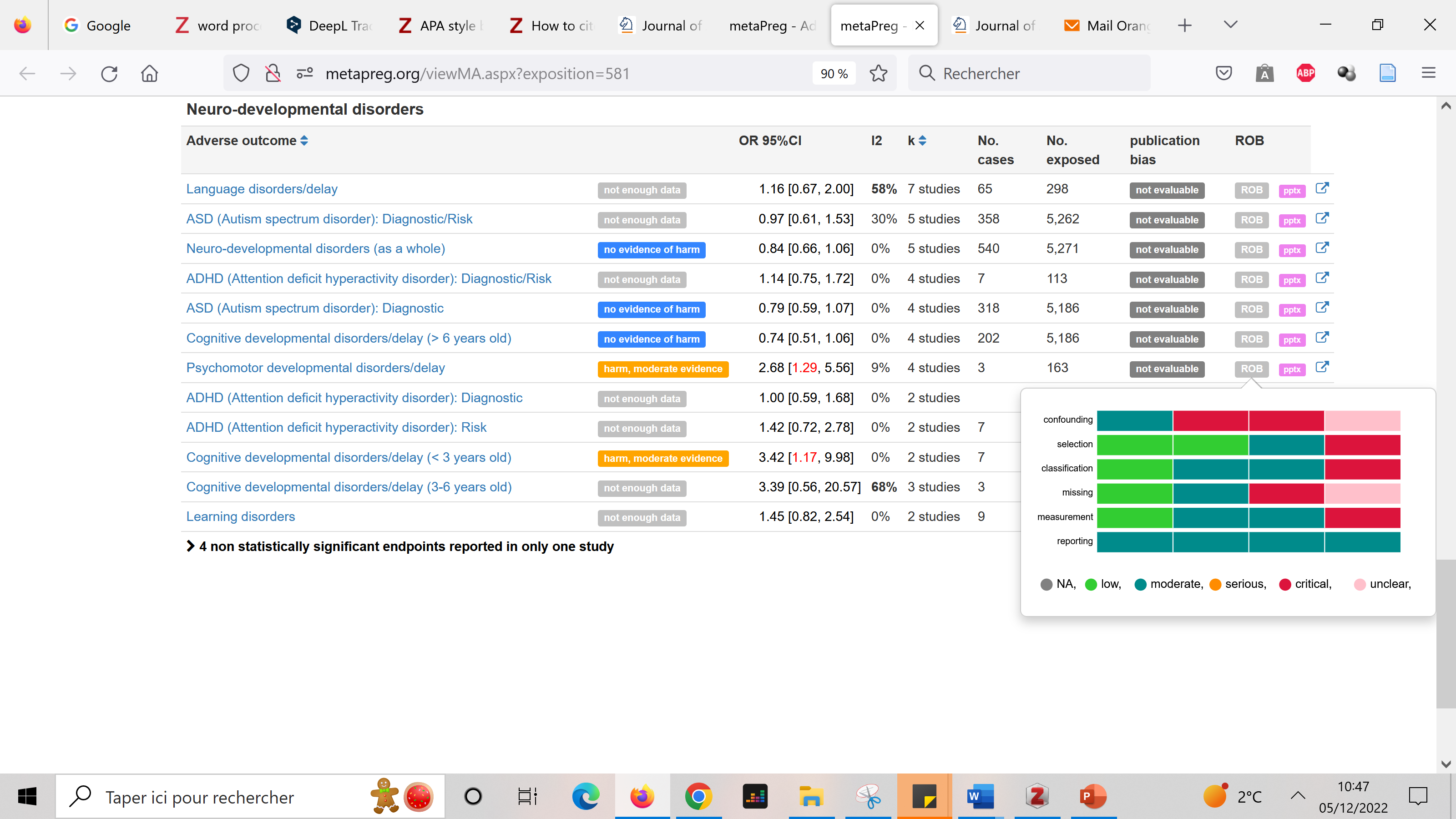

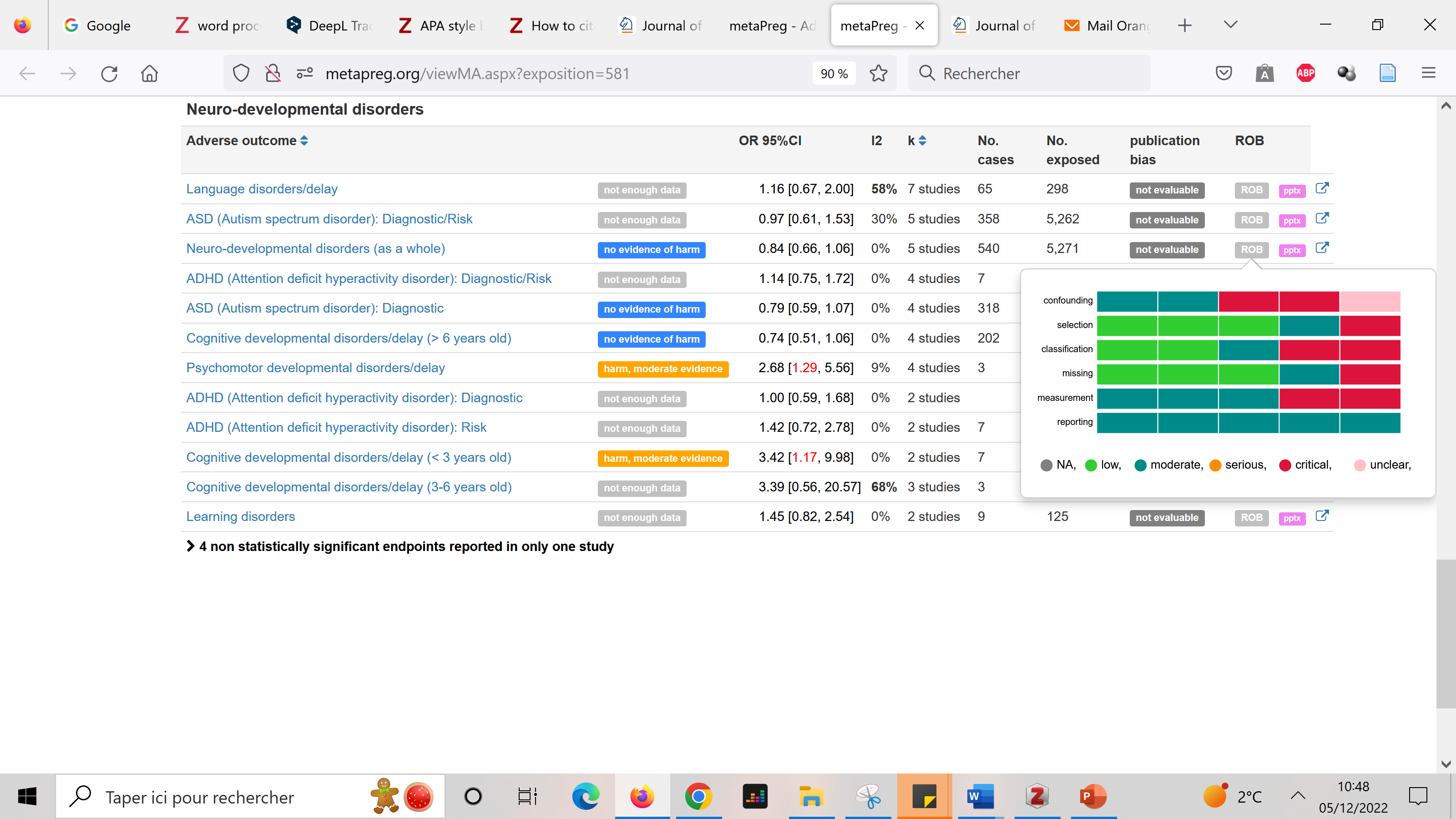


Supplement S8: MOOSE checklist.

| **Reporting Criteria** | **Reported (Yes/No)** | **Reported on Page No.** |
| --- | --- | --- |
| **Reporting of Background** |  |  |
| Problem definition | Yes | #4 |
| Hypothesis statement | Yes | #4 |
| Description of Study Outcome(s) | Yes | #6 |
| Type of exposure or intervention used | Yes | #6 |
| Type of study design used | Yes | #5-6 |
| Study population | Yes | #5 |
| **Reporting of Search Strategy** |  |  |
| Qualifications of searchers (eg, librarians and investigators) | Yes | #7 |
| Search strategy, including time period included in the synthesis and keywords | Yes | #5 and Supplement S1 |
| Effort to include all available studies, including contact with authors | Yes | #5 and 7 |
| Databases and registries searched | Yes | #5 |
| Search software used, name and version, including special features used (eg, explosion) | Yes | #5 |
| Use of hand searching (eg, reference lists of obtained articles) | Yes | #5 |
| List of citations located and those excluded, including justification | Yes | Figure 1 |
| Method for addressing articles published in languages other than  English | Yes | #5 |
| Method of handling abstracts and unpublished studies | Yes | #5 |
| Description of any contact with authors | Yes | #7 |
| **Reporting of Methods** |  |  |
| Description of relevance or appropriateness of studies assembled for assessing the hypothesis to be tested | Yes | #5 and Supplement S1 |
| Rationale for the selection and coding of data (eg, sound clinical principles or convenience) | Yes | #5 and Supplement S3 |
| Documentation of how data were classified and coded (eg, multiple raters, blinding, and interrater reliability) | Yes | #7 and 9 |
| Assessment of confounding (eg, comparability of cases and controls in studies where appropriate | Yes | #8-9 |
| **Reporting Criteria** | **Reported (Yes/No)** | **Reported on Page No.** |
| Assessment of study quality, including blinding of quality assessors; stratification or regression on possible predictors of study results | Yes | #7-8 |
| Assessment of heterogeneity | Yes | #9 |
| Description of statistical methods (eg, complete description of fixed or random effects models, justification of whether the chosen models account for predictors of study results, dose-response models, or cumulative meta-analysis) in sufficient detail to be replicated | Yes | #9-10 |
| Provision of appropriate tables and graphics | Yes | Tables and Figures |
| **Reporting of Results** |  |  |
| Table giving descriptive information for each study included | Yes | Table 1 and Supplement S3 |
| Results of sensitivity testing (eg, subgroup analysis) | Yes | Supplement S6 |
| Indication of statistical uncertainty of findings | Yes | Figure 2 and Table 2 |
| **Reporting of Discussion** |  |  |
| Quantitative assessment of bias (eg, publication bias) | Yes | Supplement S5 |
| Justification for exclusion (eg, exclusion of non–English-language citations) | Yes | Figure 1 and Supplement S4 |
| Assessment of quality of included studies | Yes | Supplement S7 |
| **Reporting of Conclusions** |  |  |
| Consideration of alternative explanations for observed results | Yes | #23-27 |
| Generalization of the conclusions (ie, appropriate for the data presented and within the domain of the literature review) | Yes | #27 |
| Guidelines for future research | Yes | #27-28 |
| Disclosure of funding source | Yes | Title page |
